# Both landscape and local factors influence plant and hexapod communities of industrial water‐abstraction sites

**DOI:** 10.1002/ece3.8365

**Published:** 2022-02-17

**Authors:** Chloé Thierry, Benoît Pisanu, Nathalie Machon

**Affiliations:** ^1^ UMS 2006 Patrimoine Naturel OFB, MNHN, CNRS Brunoy France; ^2^ Centre d'Ecologie et des Sciences de la Conservation (CESCO, UMR7204) Sorbonne Université, MNHN, CNRS Paris France

**Keywords:** agricultural practices, functional traits, graph theory, landscape connectivity, *Orthoptera*, *Rhopalocera*

## Abstract

1. At the landscape level, intensification of agriculture, fragmentation, and destruction of natural habitats are major causes of biodiversity loss that can be mitigated at small spatial scales. However, the complex relationships between human activities, landscapes, and biodiversity are poorly known. Yet, this knowledge could help private stakeholders managing seminatural areas to play a positive role in biodiversity conservation.
2. We investigated how water‐abstraction sites could sustain species diversity in vascular‐plant communities and two taxonomic groups of insect communities in a fragmented agricultural landscape.
3. Landscape‐scale variables (connectivity indices and surrounding levels of herbicide use), as well as site‐specific variables (soil type for vascular plants, floral availability for *Rhopalocera*, and low herbaceous cover for *Orthoptera*), were correlated to structural and functional metrics of species community diversity for these taxonomic groups, measured on 35 industrial sites in the Ile‐de‐France region in 2018–2019.
4. *Rhopalocera* and *Orthoptera* consisted essentially of species with a high degree of dispersal and low specialization, able to reach the habitat patches of the fragmented landscape of the study area. Sandy soil harbored more diverse vascular‐plant communities. Plant diversity was correlated to a greater abundance of *Rhopalocera* and a lower richness of *Orthoptera*.
5. Increasing landscape connectivity was related to higher abundance of plants and *Rhopalocera*, and a higher evenness index for *Orthoptera* communities. Higher levels of herbicide use were related to a decrease in the biodiversity of plants and *Rhopalocera* abundance. High levels of herbicide favored high‐dispersal generalist plants, while high levels of connectivity favored low‐dispersal plants. Specialist *Orthoptera* species were associated with low herbaceous cover and connectivity.
6. Water‐abstraction sites are valuable seminatural habitats for biodiversity. Changing intensive agricultural practices in surrounding areas would better contribute to conserving and restoring biodiversity on these sites.

## Graphical abstract

Landscape connectivity has a positive effect on plant and insect species community diversity on water‐abstraction sites; Level of herbicide treatments on crops in the surrounding landscape has a negative effect on species diversity of flora and *Rhopalocera* harbored by these industrial sites; and Improving landscape connectivity around these sites can enhance their role as seminatural habitats for biodiversity, especially in a less‐intensive agricultural context.

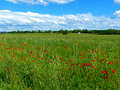

## 1 INTRODUCTION

Human activities are bringing about profound changes in land use worldwide. Among others, industrial and agricultural activities that fragment, pollute, and destroy natural habitats are responsible for the current erosion of biodiversity (Barbault, 2001; Fahrig, 2003; Pimm et al., 1995; Vitousek et al., 1997). Composition and configuration of landscapes, in particular, strongly influence dispersal, spatial distribution, and persistence of species (Beier & Noss, 1998; Debinski et al., 2001; Jeanneret et al., 2003; Mazerolle & Villard, 1999; Turner, 1989; Waldhardt, 2003). Within landscapes, connections between habitat patches are essential, as they facilitate organism dispersal, gene flow, and multiple other ecological functions (e.g., Devictor et al., 2007; Grashof‐Bokdam & Langevelde, 2005; Ricotta et al., 2000; Taylor et al., 1993). Connectivity of a landscape is defined as “the degree to which it facilitates or impedes movement along resource patches” (Taylor et al., 1993). Thus, the more connected a patch is, the richer its biodiversity should be. Identifying the main reservoirs and corridors for species movement, as well as the obstacles to the functioning of ecological continuities, is important because connections are the basis on which policies for biodiversity preservation are founded to manage territorial development (Bennett, 2003).

Nevertheless, a given landscape can be perceived as either connected or disconnected by species having different dispersal abilities (Bunn et al., 2000). Connectivity is likely influenced not only by distance between sites but also by the permeability of the inter‐site matrix (Ewers & Didham, 2006; Powney et al., 2011; Vergara, 2011), which is constituted by structures more or less easy to cross depending on the species. Various methods exist to model landscape connectivity, which can be separated into three categories: (a) those oriented toward the analysis of structural connectivity (spatial analysis of landscape components); (b) potential functional connectivity (analysis based on landscape structure and species dispersal data); or (c) actual functional connectivity (analysis based on precise knowledge of actual species movements). The graph theoretical approach, which allows modeling potential functional connectivity, offers a good trade‐off between data requirements and information provided (Calabrese & Fagan, 2004).

In European rural areas, the drastic change in agricultural practices since the second half of the 20th century has particularly affected landscape connectivity and biodiversity. Agriculture has intensified, leading to an increase in external inputs such as fertilizers and pesticides, mechanization, parcel sizes, and monoculture (Meeus, 1993; Stoate et al., 2001; Tilman et al., 2002). Thus, by fragmenting, destroying, and polluting seminatural habitats, these practices have contributed to the homogenization of landscapes (Benton et al., 2003; Foley et al., 2005). Numerous studies have shown the impacts of agricultural intensification on many taxa (e.g., Cherrill, 2010; Ekroos et al., 2010; Hutton & Giller, 2003; Krebs et al., 1999), and pesticides are in particular blamed for their persistent negative effects (Geiger et al., 2010).

In these fragmented landscapes, seminatural elements such as hedgerows, woodlands, permanent meadows, grassy strips, and ditches make the landscape more heterogeneous and more favorable to biodiversity (Benton et al., 2003). Their richness, diversity, and composition of communities depend on the regional pool of species and on local and landscape factors, which interact in complex ways. These elements are known to play a role as habitats, refuges, or corridors for many species, depending on their management, structure, and composition within their landscape context (Concepción et al., 2012; Reeder et al., 2005; Rodríguez & Bustamante, 2008; Villemey et al., 2015). Industrial sites often contribute to the fragmentation and destruction of habitats, and can be a source of various types of pollution (Jones et al., 2015; Krannich & Albrecht, 1995; Zeiss & Atwater, 1987). However, in some cases, they consist of seminatural habitats and, depending on the landscape context, they can therefore play a key role as stepping stones, refuges, or habitats for biodiversity, particularly when they are managed ecologically (Serret et al., 2014; Snep et al., 2009; Thuillier, 2020). Among industrial sites, water‐abstraction sites, which provide drinking water to the population, are found all over the world. They consist of extracting water from a source and transporting it to a distribution network or to a treatment facility. Although their characteristics may vary according to the country and the type of source (groundwater or surface water), they are generally small sites that include a catchment and a protective perimeter to prevent physical damage and the direct introduction of toxic substances into the water or soil. For this reason, water‐abstraction sites are often covered with seminatural vegetation and are relevant to study relationships between local and landscape characteristics and biodiversity. Indeed, the studies on these sites mainly highlight their impacts on aquatic biodiversity and stream function, but there are gaps in knowledge about their potential role in maintaining terrestrial biodiversity (Arroita et al., 2017; Brooks et al., 2015; Pardo & García, 2016).

We conducted our study in an agricultural landscape mainly composed of croplands and including water‐abstraction sites. Our questions were the following: What is the species richness and composition of seminatural habitats in water‐abstraction sites? What is the relative importance of the local environmental conditions, landscape connectivity, and the management of the surrounding croplands for the flora and fauna of these sites?

To answer these questions, we studied three taxonomic groups, namely vascular flora, *Rhopalocera* (butterflies), and *Orthoptera* (crickets, grasshoppers, and locusts). These were chosen because they are regarded as ecological indicators of habitat quality and landscape composition (Bazelet & Samways, 2011; Pe’er & Settele, 2008; Terwayet Bayouli et al., 2021) with dispersal capacities consistent with the scale of the study (Defaut & Morichon, 2015; Hernández et al., 2015; Stevens et al., 2010). In addition, these groups have different movement characteristics, which make them interesting for testing the impacts of local and landscape factors. Plants form the basis of the ecosystem and are resources for the two other taxa. While butterflies are rather mobile and relatively specialized, *Orthoptera* species are more sedentary and less selective (Marini et al., 2009). As several studies have shown the potential of industrial sites for biodiversity when managed in an ecological way (Serret et al., 2014; Snep et al., 2009; Thuillier, 2020), we expected that the studied water‐abstraction sites, which are subject to a biodiversity‐friendly late mowing per year, will host many species. Furthermore, we assume an influence of both local and landscape factors, with greater importance of local factors for all taxa studied, especially for the flora that is sessile (even if propagules can disperse over long distances) (Marini et al., 2008; Pöyry et al., 2009; Sutcliffe et al., 2015).

## 2 METHODS

### 2.1 Study region and sites

**FIGURE 1:**
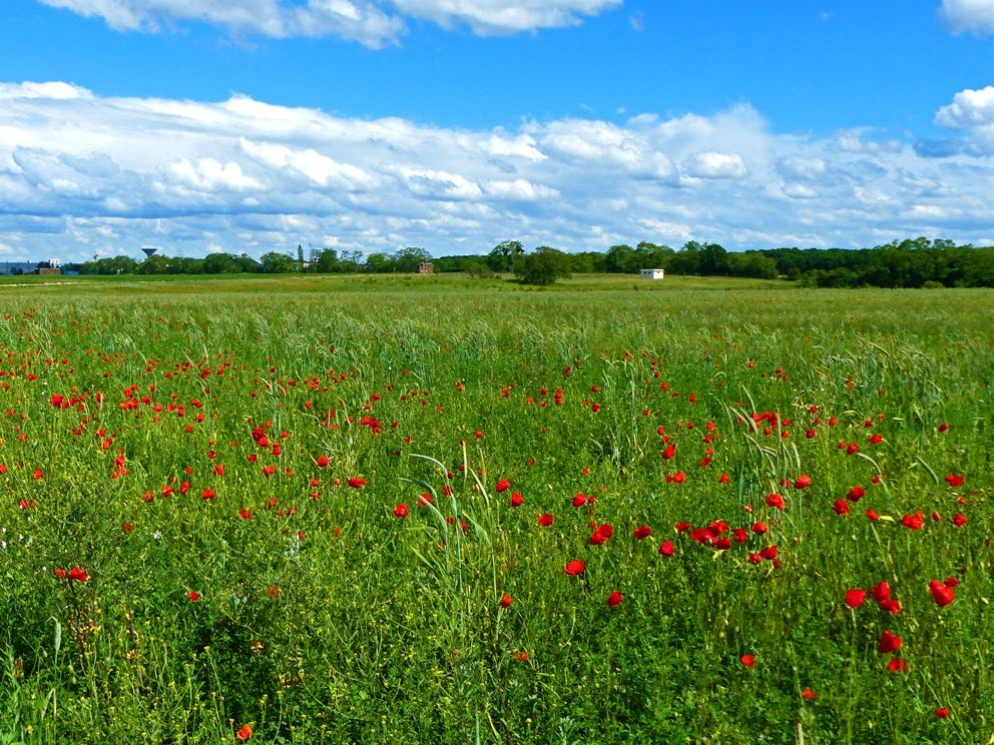
Water‐abstraction site and agricultural fields (© Chloé Thierry)

Our study focused on an area of 13 × 18 km in the Yvelines Department in France (west of Paris), between the cities of Mantes‐la‐Jolie and Les Mureaux (Supplementary Material). This area is marked by increasing urban development along the Seine River and is dominated by agricultural land with some seminatural habitats. Thirty‐five water‐abstraction sites were studied in this area, ranging in size from 0.1 to 1 ha and regularly distributed in an essentially agricultural matrix (mostly field crops and vegetable cropping) (Figure 1). All created between 1960 and 1970, these small industrial sites are fenced and include a groundwater catchment, covered by a concrete base and/or a building, and a protective perimeter where the use of pesticides is prohibited. They mainly consist of open areas, dry grasslands, or mesophilic‐to‐meso‐hygrophilic meadows, managed with one late mowing per year.

### 2.2 Biodiversity sampling

#### 2.2.1 Flora

We used the Vigie‐Flore protocol (www.vigie‐flore.fr) to inventory the plant species present on the 35 water‐abstraction sites. We visited each site in mid‐June 2018 to inventory a ten‐square‐meter plot, divided into 10 quadrats of 1 m^2^. In each quadrat, a presence–absence list of all plant species was produced. For each species, we calculated the abundance as the number of quadrats in which it was present.

#### 2.2.2 Rhopalocera

We recorded *Rhopalocera* species by visiting the 35 sites four times in 2018, during the periods (May, June, July, and August) of maximum activity and density of the species and under favorable weather conditions. We used the STERF (Temporal Monitoring of *Rhopalocera* in France) protocol (Manil & Henry, 2007) in which butterflies are counted and identified by moving along one transect per site over a period of 10 min. For each visit, we noted the cover for flowering plants on the site.

#### 2.2.3 Orthoptera

To study *Orthoptera*, we visited 34 water‐abstraction sites (one of the sites was no longer accessible) in early August 2019, that is, the period when the adults were the most numerous and active. We used the protocol described in Lacoeuilhe et al. (2020), based on the linear abundance index (LAI) and the method used by Voisin (1986), which consists of walking along transects 20 m long and noting the number of specimens fleeing in front of the observer's footsteps over a strip approximately one meter wide (Jaulin, 2009). Two transects per site were inventoried under good weather conditions. We also noted the cover (%) for three classes of herbaceous vegetation for each site: low (<20 cm), medium (20–40 cm), and high (>40 cm).

**FIGURE 2:**
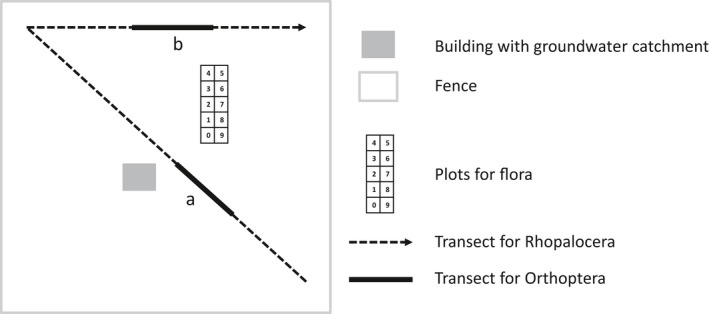
Sampling plan implemented in the 35 water‐abstraction sites studied

The position of the quadrats and transects on the sites is indicated in Figure 2.

### 2.3 Species traits

We studied different diversity dimensions including functional diversity, which allows a better understanding of the different aspects of the functioning of an ecosystem such as its dynamics or its stability (Goswami et al., 2016).

To examine whether community characteristics could be linked to functional traits of species constituting them, we calculated the community‐weighted mean trait values, that is, the mean values of traits of the species weighted by their abundance in the community, for different selected traits (Garnier et al., 2004). For the vascular flora, we used the maximum seed‐releasing height drawn from the LEDA Traitbase (Kleyer et al., 2008) as a dispersal metric as proposed by Thomson et al. (2011). We also used for each species the species specialization index θ_wb_, as calculated by Mobaied et al. (2015) who used Whittaker׳s beta (1960) on an independent database:

$$\theta_{\text{wb}}=\gamma /\mu \left(\alpha \right)$$

where *γ* is the cumulative number of species over all plots containing a given species, and *µ*(*α*) is mean plot species richness.

Finally, we took into account the species dependence on insect pollination by combining pollen‐vector information from CATMINAT (Julve, 1998), Ecoflora (Fitter & Peat, 1994), and BiolFlor (Klotz & Durka, 2002; Kühn et al., 2004) traitbases according to the method described in Martin (2018).

For *Rhopalocera* species, we used their dispersal (1–3) and specialization (1–4) classes as described in the DuPont (2015) database.

For *Orthoptera*, we also used three classes of dispersal and two classes of specialization according to Reinhardt et al. (2005) and Marini et al. (2010). When the specialization class was not available for a species, we deduced it from their habitat descriptions in Bellman and Lucquet (2009), considering species that require specific moisture and vegetation conditions as specialists, and undemanding mesophilic species as generalists. For individuals identified at the genus level, we assigned a trait value if all species within the genus shared the same trait. If not, no value was assigned. All observations with unknown traits were removed from the analyses (Table 1).

**TABLE 1:** Description and statistics of the traits used for the 3 taxonomic groups studied

Trait name	Description	Mean	Range	Number of species with information/number of inventoried species	Percentage of observations with information	Traitbases and sources
Flora						
Dispersal	Maximum seed‐releasing height as a proxy for dispersal (increases with dispersal)	1	0.1–25	142/147	94.4	LEDA from Kleyer et al. (2008); Thomson et al. (2011)
Specialization	Index *θ*_wb_, calculated using species co‐occurrence data (decreases with specialization)	17.3	8.3–24.3	135/147	90.5	Whittaker (1960); Mobaied et al. (2015)
Pollination dependence	Percentage of times “insects” appears as a pollen vector for a given species across various databases	56.1	0–100	146/147	94.4	CATMINAT from Julve (1998); Ecoflora from Fitter and Peat (1994); Biolflor from Klotz and Durka (2002) and Kühn et al. (2004); Martin (2018)
Rhopalocera						
Dispersal	Three classes, based on movements between two patches of favorable habitat 1: Low dispersal (majority of movements are within the patch or an adjacent patch) 2: Medium dispersal (majority of movements are across the habitat ecocomplex) 3: High dispersal (individuals’ movements allow the visit of several ecocomplexes with favorable habitats)	None	None	32/32	99.2	Dupont (2015)
Specialization	Four classes, based on the optimal habitat of the caterpillar 1: Generalist species whose caterpillars grow in many types of habitat 2: Moderately generalist species whose caterpillars grow mainly in the associated habitat 3: Specialist species whose caterpillars grow mainly in the associated habitat 4: Specialist species with a very localized distribution	None	None	32/32	99.2	Dupont (2015)
Orthoptera						
Dispersal	Three classes, based on the wing development of the adults, individual movement in population studies, and long‐term observations of local and regional colonization dynamic 1: low‐mobile species 2: moderate‐mobile species 3: high‐mobile species	None	None	17/17	83.2	Reinhardt et al. (2005); Marini et al. (2010)
Specialization	Two classes, based on moisture preferences 0: Generalist species (mesophilic species) 1: Specialist species (xerothermic and hygrophilic species)	None	None	17/17	72.4	Reinhardt et al. (2005); Bellman and Lucquet (2009)

### 2.4 Connectivity metrics

We compiled the GIS databases of the study area, as described in Thierry et al. (2020), in order to obtain the land‐use map. All these data elements were combined into a single raster layer with a resolution of 2 m in order to highlight linear elements or small constructions that can impact the movement of species.

To estimate the connectivity of the study sites, we used graph theory (Urban & Keitt, 2001) with Graphab software (Foltête et al., 2012) and cost distances. We allocated costs to each land‐use category according to its resistance to movement for each species type, based on ecological literature and opinions of experts on the taxa studied (see Acknowledgments). These costs are as follows for *Rhopalocera* and *Orthoptera* in herbaceous environments:

1 for their habitat patches,
10 for favorable elements,
100 for unfavorable elements, and
1000 for elements considered as "barriers" (Supplementary Material).

In the case of small habitat mosaics, we pooled them into a single habitat and assigned the average value of the composite habitats. For plant species, given that dispersal in herbaceous environments is complex and largely driven by wind (57.4% of the species studied are partially or totally wind‐dispersed), we considered only forests and buildings over 15 m tall as barrier elements (cost of 1000) and all herbaceous environments as habitat patches (cost of 1), and the other land‐use categories (e.g., agricultural fields, shrubs, roads, and other artificial spaces) were assigned a cost of 10.

We designed planar, non‐thresholded graphs and used the probability of connectivity (PC) index, which is defined as “the probability that two organisms randomly placed within the landscape fall into habitat areas that are reachable from each other” (Saura & Pascual‐Hortal, 2007). The PC was calculated as follows:

$$\text{PC}=\frac{\sum_{i=1}^{n}\sum_{j=1}^{n}a_{i}a_{j}p_{\mathrm{ij}}}{A^{2}}$$

where *p_ij_* is the maximum probability of movement between the patches *i* and *j*, *a_i_* and *a_j_* are the areas of the patches *i* and *j*, *A* is the total area of the study zone, and *n* is the total number of patches.

An exponential function can be used to calculate *p_ij_* as follows:

$$p_{\mathrm{ij}}=e^{‐kdij}$$

Where *dij* is the least‐cost distance between the patches *i* and *j*, and *k* (0 < *k* < 1) expresses the reduction in dispersal probabilities resulting from this exponential function (Saura & Pascual‐Hortal, 2007).

For *Rhopalocera* and *Orthoptera*, we used dispersal distances of 100 m for poorly mobile species and 300 m for moderately mobile ones (Defaut & Morichon, 2015; Olivier et al., 2016; Stevens et al., 2013). For Flora, we chose 150 m for poorly mobile species and 500 m for moderately mobile ones (Hernández et al., 2015; Rambaud, 2018). Given the scale of the study, it was not relevant to consider highly mobile species.

In order to obtain a connectivity value for each study site, we assigned to each the dPC value of the habitat patch in which they were located, ranked by their contribution to overall landscape connectivity according to the PC index (Keitt et al., 1997; Urban & Keitt, 2001; Pascual‐Hortal and Saura, 2006; Rae et al., 2007):

$$\text{dPC}=\frac{\text{PC}‐\text{P}{\text{C}}^{\prime }}{\text{PC}}\times 100$$

where PC is the value when the landscape element is present in the landscape, and PC' is the value after removal of that landscape element (e.g., following the loss of a habitat patch) (Supplementary Material).

### 2.5 Agricultural effects through herbicide use

We used the treatment frequency indices (TFIs) of herbicides available for the agricultural land around the sites. We calculated an average TFI based on the amount of areas treated within 100‐m and 300‐m buffer zones (data from Ile‐de‐France Interdepartmental Chamber of Agriculture, 2015). We chose these distances to represent the landscape around the study sites while avoiding too much overlap. NA (not available) was assigned to sites with information available for less than half of the surrounding land (three sites having buffer zones with a radius of 300 m, and four having buffer zones with a radius of 100 m).

### 2.6 Statistical analyses

For each taxon, we used linear models with a Gaussian error. In order to avoid model overfitting, we retained only three explanatory variables per model. We divided the variables into three categories (herbicide treatments at the landscape level, landscape connectivity, and local variables) and selected one variable per category among the least correlated ones (Table 2). We used the following structure: Response variable ~ landscape explanatory variable 1 (herbicides) + landscape explanatory variable 2 (connectivity) + local explanatory variable 3.

**TABLE 2:** Descriptive statistics of the explanatory variables considered for each taxon. Variables in bold are those that have been retained, the others having been excluded because of collinearity (correlation coefficient >0.3)

Scale	Name	Description and unit	Mean	SD	Min	Max
Flora						
Landscape	Herbicide treatment	Average treatment frequency indices for herbicides within a radius of 100 m	0.44	0.62	0	2.09
		**Average treatment frequency indices for herbicides within a radius of 300 m**	**0.41**	**0.50**	**0**	**1.82**
	Connectivity	dPC calculated for flora with dispersal distances of 150 m	3.37e−4	9.03e−4	1.19e−6	4.08e−3
		**dPC calculated for flora with dispersal distances of 500 m**	**4.89e−4**	**1.10e−3**	**1.10e−5**	**4.82e−3**
		dPC calculated for Rhopalocera with dispersal distances of 100 m (only when pollination dependence is used as the response variable)	2.70e−4	1.09e−3	1.29e−6	6.49e−3
		**dPC calculated for Rhopalocera with dispersal distances of 300 m (only when pollination dependence is used as the response variable)**	**3.17e−4**	**1.29e−3**	**2.09e−6**	**7.70e−3**
Local	Soil type	**Qualitative variable, divided into 2 categories: clay vs. sandy soil**	**None**	**None**	**None**	**None**
Rhopalocera						
Landscape	Herbicide treatment	Average treatment frequency indices for herbicides within a radius of 100 m	0.44	0.62	0	2.09
		**Average treatment frequency indices for herbicides within a radius of 300 m**	**0.41**	**0.50**	**0**	**1.82**
	Connectivity	dPC calculated for Rhopalocera with dispersal distances of 100 m	2.70e−4	1.09e−3	1.29e−6	6.49e−3
		**dPC calculated for Rhopalocera with dispersal distances of 300 m**	**3.17e−4**	**1.29e−3**	**2.09e−6**	**7.70e−3**
Local	Flowering‐plant availability	**Average cover for flowering plants over the 4 visits (%)**	**12.89**	**8.80**	**0.75**	**31.25**
Orthoptera						
Landscape	Herbicide treatment	**Average treatment frequency indices for herbicides within a radius of 100 m**	**0.44**	**0.62**	**0**	**2.09**
		Average treatment frequency indices for herbicides within a radius of 300 m	0.41	0.50	0	1.82
	Connectivity	dPC calculated for Orthoptera with dispersal distances of 100 m	3.45e−4	1.49e−3	2.63e−6	8.89e−3
		**dPC calculated for Orthoptera with dispersal distances of 300 m**	**4.16e−4**	**1.57e−3**	**2.22e−6**	**9.40e−3**
Local	Moisture	Semiquantitative variable based on site habitat vegetation, divided into three categories: 1 (xerophilous), 2 (meso‐xerophilous), 3 (meso‐hygrophilous)	None	None	None	None
	Vegetation height	**Low herbaceous cover (<20 cm) (%)**	12.06	16.63	0	70
		High herbaceous cover (>40 cm) (%)	58.71	26.88	0	95

For plants, we analyzed the influence of the level of herbicide treatment within a radius of 300 m around sampled sites, site connectivity (dPC) modeled for plants with a dispersal distance of 500 m, and soil type, on the variation in species richness, total abundance (i.e., number of quadrats in which species were recorded), evenness, dispersal, and specialization (Table 1). We also studied the influence of the same variables on pollination dependence, but using site connectivity (dPC) modeled for *Rhopalocera* with a dispersal distance of 300 m. Indeed, *Rhopalocera* play an important role in pollination, and the results of connectivity modeling for this taxonomic group are likely to represent connectivity for other pollinators using the same environments. Evenness was calculated using Pielou's evenness index (1966).

For *Rhopalocera*, we analyzed the influence of the level of herbicide treatment within a radius of 300 m around sampled sites, site connectivity (dPC) modeled for *Rhopalocera* with a dispersal distance of 300 m, and flowering‐plant availability, on the variation in species richness, total abundance (total number of individuals observed), evenness, dispersal, and specialization.

For *Orthoptera*, we analyzed the influence of the level of herbicide treatment within a radius of 100 m around sampled sites, site connectivity (dPC) modeled for *Orthoptera* with a dispersal distance of 300 m, and low herbaceous cover (<20 cm), on the same variables as for *Rhopalocera*.

Full raw data and all statistical analyses are detailed in the Supplementary Material. More specifically, continuous explanatory variables were scaled to improve coefficient interpretation (Schielzeth, 2010). The absence of collinearity was graphically checked, and all variables with a correlation coefficient >0.3 were excluded (Zuur et al., 2009, 2010) (Table 2). For this reason, the variables selected for each taxon are not all based on the same distances. Variance homogeneity, the absence of influential points, and the absence of spatial autocorrelation were graphically checked (Zuur et al., 2009, 2010; Supplementary Material). To study relations between taxa, we computed Pearson's correlation coefficients (Sokal & Rohlf, 1995) on the different community species‐diversity metrics. Mean values are followed by a standard error value throughout the manuscript, unless otherwise stated.

## 3 RESULTS

During our floristic inventories, we observed a total of 147 plant species on the sites (10.3% of regional species, 14 of which are rare or endangered), with an average of 21 species per site (min = 8, max = 35). The abundance averaged 112 and ranged from 34 to 203 per site (3930 in total, with 95% identified to the species level and 5% to the genus level). Over four visits, we inventoried a total of 32 species of *Rhopalocera* on the 35 abstraction sites (28.6% of regional species, 3 of which are rare or endangered), with an average of 6 species per site (min = 1, max = 12). We counted 997 individuals (94% identified to the species, 5% to the genus level, 1% non‐identified), with an average of 29 observed per site (min = 1, max = 86). The total number of *Orthoptera* species recorded was 17 (25.0% of regional species, 4 of which are rare or endangered), with 692 individuals counted (72% identified to the species, 12% to the genus level, and 16% non‐identified). The average richness per site was 5 (min = 1, max = 7), and the average abundance was 20 (min = 6, max = 49).

### 3.1 Flora

**FIGURE 3:**
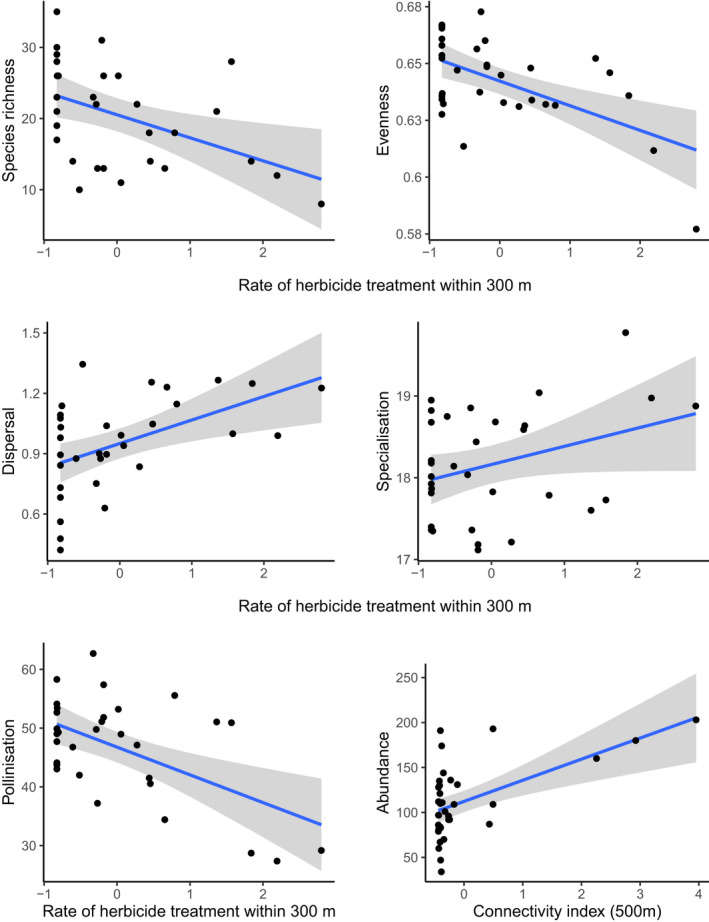
Relationships between herbicide treatment levels within a 300‐m radius around sites on vascular‐plant species richness, evenness and functional metrics, abundance, and the floral connectivity index, from 32 sampled sites. All explanatory variables are scaled

Species richness, total abundance, and evenness indices for vascular‐plant communities, as well as the community‐pollination metric, all decreased with increasing levels of herbicide treatments within a radius of 300 m around sampled sites (Table 3; Figure 3). The community‐dispersal metric increased with levels of herbicides, while specialization decreased (i.e., the specialization index increased) (Table 3; Figure 3). Compared to sites with sandy soils, those with clay soils harbored vascular‐plant communities with fewer species on average (clay: *n* = 12 sites, 15 ± 2 species; sandy: *n* = 20, 24 ± 1) and less abundance in plants (clay: *n* = 12, 83 ± 8; sandy: *n* = 20, 132 ± 9); however, there was no major difference in the evenness indices (Table 3; clay: *n* = 12, 0.634 ± 0.007; sandy: *n* = 20, 0.647 ± 0.003). Vascular‐plant abundance increased with increasing levels of the floral connectivity index modeled for species with a dispersal distance of 500 m, while the community‐dispersal metric decreased with the increasing floral connectivity index (Table 3; Figure 3).

**TABLE 3:** Results of linear models exploring the influence of the connectivity index, herbicide treatments and soil types for vascular plants, floral availability for *Rhopalocera*, and cover for low herbaceous species, on community diversity measures from 35 water‐abstraction sites sampled between 2018 and 2019

Taxonomic group	Vascular plants		*Rhopalocera*		*Orthoptera*	
Sources of variation	*β* ± *SE*	*p*	*β* ± *SE*	*p*	*β* ± *SE*	*p*
Species richness						
Intercept	16.57 ± 1.58	<.001	6.36 ± 0.44	<.001	4.54 ± 0.30	<.001
Connectivity	1.77 ± 0.98^1^	.081	−0.08 ± 0.44^2^	.857	−0.38 ± 0.31^2^	.236
Herbicide treatment	−**2.08** **± 0.99^2^**	.**045**	−0.58 ± 0.47^2^	.223	**0.92 ± 0.32** ^3^	.**009**
Soil type _Sandy versus Clay_	**6.30 ± 2.04**	.**004**	–	–	–	–
Floral availability	–	–	0.50 ± 0.48	.304	–	–
Low herbaceous cover	–	–	–	–	−0.18 ± 0.31	.569
Abundance						
Intercept	92.35 ± 8.42	<.001	27.20 ± 3.19	<.001	20.21 ± 1.99	<.001
Connectivity	**15.13 ± 5.24** ^1^	.**007**	**9.27 ± 3.14** ^2^	.**006**	−3.10 ± 2.13^2^	.160
Herbicide treatment	−**15.04** **± 5.31^2^**	.**008**	−**8.84** **± 3.37** ^2^	.**014**	−0.53 ± 2.22^3^	.814
Soil type _Sandy versus Clay_	**33.72 ± 10.88**	.**004**	–	–	–	–
Floral availability	–	–	2.67 ± 3.45	.445	–	–
Low herbaceous cover	–	–	–	–	0.63 ± 2.16	.772
Evenness						
Intercept	–0.64 ± 0.01	<.001	0.61 ± 0.01	<.001	0.55 ± 0.02	<.001
Connectivity	<0.01^1^	.451	0.01 ± 0.01^2^	.521	**0.05 ± 0.02** ^2^	.**020**
Herbicide treatment	−**0.01** **± 0.00^2^**	.**004**	0.01 ± 0.01^2^	.289	0.03 ± 0.02^3^	.267
Soil type _Sandy versus Clay_	0.01 ± 0.05	.257	–	–	–	–
Floral availability	–	–	−0.01 ± 0.01	.302	–	–
Low herbaceous cover	–	–	–	–	−0.01 ± 0.02	.550
Dispersal						
Intercept	1.01 ± 0.05	<.001	2.74 ± 0.05	<.001	n.c.	–
Connectivity	−**0.09** **± 0.03^1^**	.**017**	−0.02 ± 0.05^2^	.731	n.c.	–
Herbicide treatment	**0.08 ± 0.03^2^**	.**022**	−0.07 ± 0.05^2^	.144	n.c.	–
Soil type _Sandy versus Clay_	−0.09 ± 0.07	.208	–	–	–	–
Floral availability	–	–	0.05 ± 0.05	.316	–	–
Low herbaceous cover	–	–	–	–	n.c.	–
Specialization						
Intercept	18.13 ± 0.20	<.001	1.13 ± 0.21	<.001	0.18 ± 0.04	<.001
Connectivity	0.12 ± 0.12^1^	.340	0.04 ± 0.03^2^	.168	**0.08 ± 0.04^2^**	.**040**
Herbicide treatment	**0.26 ± 0.12^2^**	.**043**	−0.03 ± 0.03^2^	.244	−0.01 ± 0.04^3^	.855
Soil type _Sandy versus Clay_	0.05 ± 0.25	.856	–	–	–	–
Floral availability	–	–	0.02 ± 0.03	.468	–	–
Low herbaceous cover	–	–	–	–	**0.16 ± 0.04**	.**001**
Pollination dependence						
Intercept	45.48 ± 2.17	<.001	–	–	–	–
*Rhopalocera* connectivity	0.30 ± 1.30^2^	.820	–	–	–	–
Herbicide treatment	**−4.46 ± 1.37^2^**	.**003**	–	–	–	–
Soil type _Sandy versus Clay_	2.05 ± 2.78	.466	–	–	–	–

Note

Calculated for distances of **^1^** 500 m, **^2^** 300 m, and **^3^** 100 m (see the Material and Methods section); n.c.: not calculated; significant results at the 0.05 level are in bold type.

### 3.2 Rhopalocera

**FIGURE 4:**
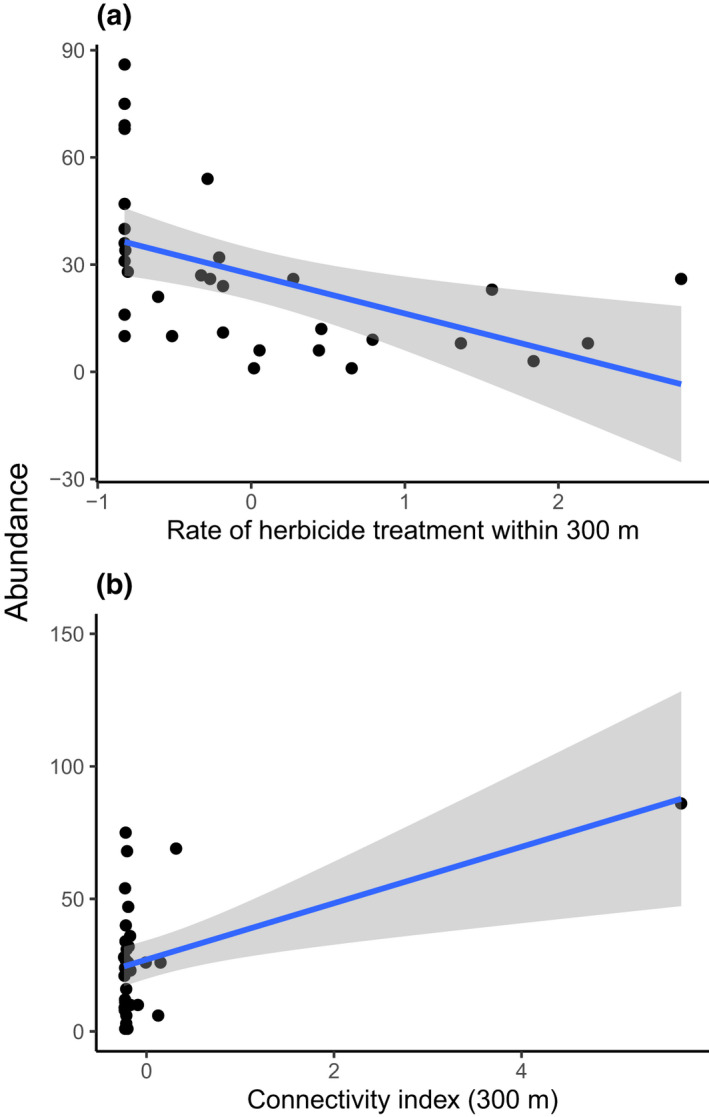
Relationships between Rhopalocera species abundance and (a) the level of herbicide treatments within a 300‐m radius and (b) the connectivity index for Rhopalocera species with a dispersal distance of 300 m, from 32 sampled sites. All explanatory variables are scaled

Total abundance of *Rhopalocera* seemed to be influenced not only by the connectivity metric calculated for species with a dispersal distance of 300 m, but also by the level of herbicide treatment within a radius 300 m around sites (Table 3). *Rhopalocera* abundance increased with increasing levels of connectivity (Figure 4a) and decreased with increasing levels of pesticide treatments (Figure 4b). Species richness, evenness, and the community‐dispersal and community‐specialization metrics did not vary with the connectivity index, pesticide treatment levels, or floral availability (Table 3).

### 3.3 Orthoptera

**FIGURE 5:**
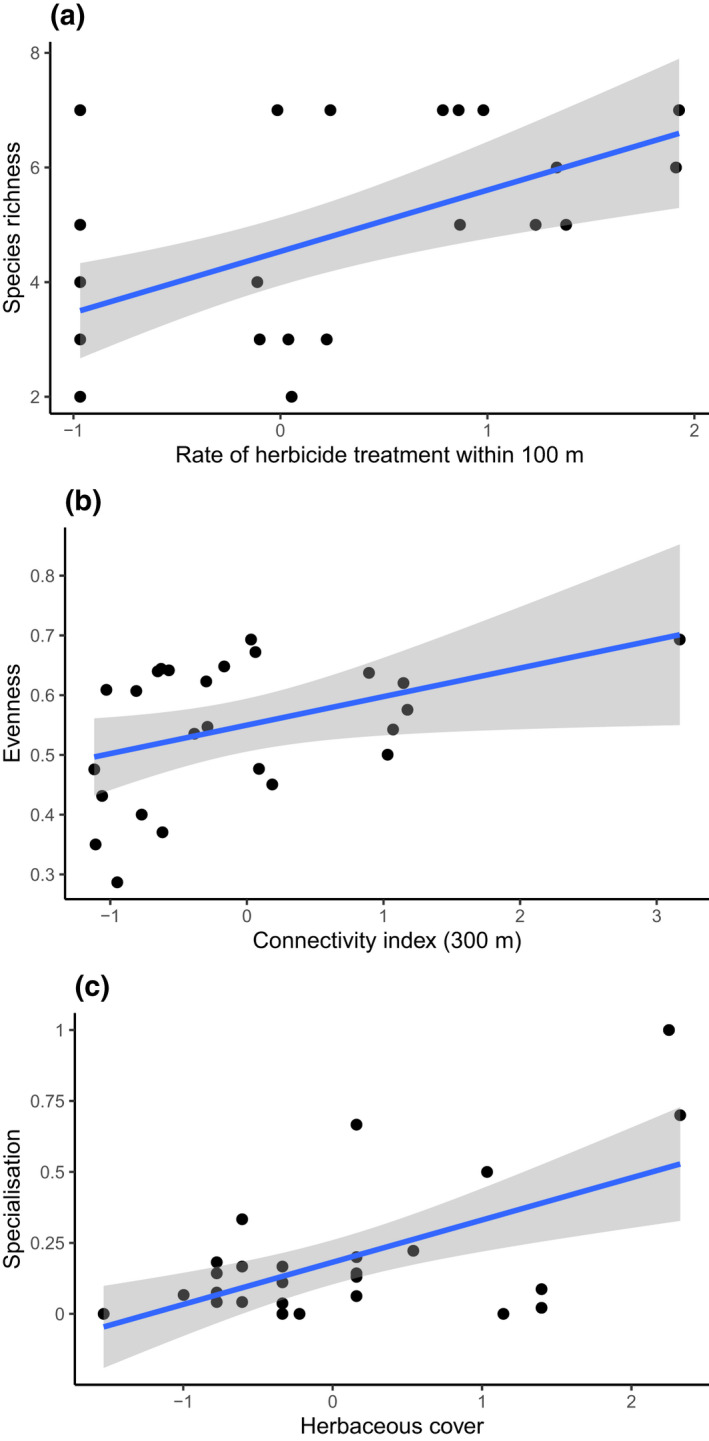
Relationships between (a) species richness and the level of herbicide treatments within a radius of 100 m, (b) evenness, and (c) community specialization and low herbaceous cover, for *Orthoptera* species communities from 29 sampled sites. All explanatory variables are scaled

*Orthoptera* species richness increased with increasing levels of herbicide treatments within a radius of 100 m around sampled sites (Table 3; Figure 5), and the evenness index increased with increasing levels of the *Orthoptera* connectivity index modeled for species with a dispersal distance of 300 m (Table 3; Figure 5). Community‐specialization metrics increased when low herbaceous cover and connectivity increase (Table 3; Figure 5). Total abundance did not vary with the connectivity index, pesticide treatment levels, or low herbaceous cover (Table 3).

### 3.4 Correlations between taxa

The abundance of *Rhopalocera* correlated positively with the species richness and abundance of plants. Conversely, we observed a negative correlation between species richness in *Orthoptera* and all measures of plant diversity. The abundance of *Orthoptera* and the species richness of *Rhopalocera* correlated positively, as well as the species richness of *Orthoptera* and the evenness of *Rhopalocera*, while the species richness of *Orthoptera* and the abundance of *Rhopalocera* correlated negatively (Table 4).

**TABLE 4:** Pearson's correlation coefficients calculated between communities species‐diversity measures (Sr: species richness; Ab: abundance; Ev: evenness index; CWMs: specialization; CWMd: dispersal; and CWMdp: pollination dependence)

	Vascular plants						Rhopalocera					Orthoptera				
	Sr	Ab	E	CWMs	CWMd	CWMdp	Sr	Ab	E	CWMs	CWMd	Sr	Ab	E	CWMs	CWMd
Vascular plants (*n* = 35, *r*_Pearson_ ≥ 0.42)																
*Sr*	–															
*Ab*	**0.90**	–														
*E*	**0.50**	**0.66**	–													
*CWMs*	−0.29	−0.17	−0.29	–												
*CWMd*	**−0.55**	**0.75**	**0.48**	0.06	–											
*CWMdp*	**0.55**	**0.47**	**0.45**	**−0.52**	−0.37	–										
Rhopalocera (*n* = 32, *r*_Pearson_ ≥ 0.45)																
*Sr*	0.22	0.10	0.06	−0.43	−0.13	0.16	–									
*Ab*	0.43	0.37	0.28	−0.26	−0.37	0.33	0.40	–								
*E*	−0.33	−0.31	<0.01	−0.06	0.28	−0.16	−0.25	**−0.47**	–							
*CWMs*	0.25	0.18	<0.01	−0.41	−0.12	0.19	**0.49**	0.41	<0.01	–						
*CWMd*	<0.01	0.06	0.20	−0.13	−0.12	0.39	**0.46**	0.28	−0.30	0.07	–					
Orthoptera (*n* = 28, *r*_Pearson_ ≥ 0.46)																
*Sr*	**−0.59**	**−0.70**	**−0.54**	0.20	**0.67**	−0.45	−0.07	**−0.65**	0.43	−0.07	−0.26	–				
*Ab*	−0.12	−0.23	−0.26	−0.32	0.15	0.22	0.40	−0.16	0.21	0.27	0.19	**0.48**	–			
*E*	<0.01	<0.01	<0.01	0.19	0.13	**−0.46**	−0.29	<0.01	0.09	0.07	−0.33	0.04	**−0.55**	–		
*CWMs*	**0.52**	**0.59**	0.31	0.23	**−0.56**	0.08	−0.14	0.20	0.10	0.13	−0.11	−0.38	−0.32	0.21	–	
*CWMd*	−0.27	−0.15	−0.09	−0.18	0.12	−0.08	−0.23	−0.44	**0.49**	<0.05	−0.12	0.18	0.18	0.07	−0.32	–

Note

Significant coefficients at the 0.01 level are in bold type.

## 4 DISCUSSION

We found that the level of herbicide treatments on crops in the surrounding landscape affected species diversity of flora and *Rhopalocera* on the water‐abstraction sites we studied. Increasing landscape connectivity seemed to favor more diverse communities of *Rhopalocera* and *Orthoptera* species.

### 4.1 Effects of herbicide treatments

Herbicide use on agricultural land around abstraction sites (not treated) seemed to influence all the taxa we studied. As expected (Geiger et al., 2010), vascular plants seemed to be the most impacted in terms of all the diversity measures. This result shows how herbicides probably affect all types of plants within a radius of at least 300 m. It could be a direct effect of herbicides through the spill of chemicals from landscape, or an indirect one if herbicides suppress species that cannot reach the plots via dispersal. In our study as in others, herbicide use also correlated with a lower abundance of *Rhopalocera*. The impact of herbicides on insects is usually attributed to limited amounts of available resources (e.g., Muratet & Fontaine, 2015), which could be the case for this study. The effect of crop types could also play a role as cereal crops were more likely to be treated. Cereal crops are generally not dependent on pollinating insects compared with fruit, vegetable, or legume crops (Schneider & Huyghe, 2015). More surprisingly, the richness of *Orthoptera* on the sites tended to be higher when herbicides were used around them. Nevertheless, sites near a landscape where herbicides were used were generally wetter with more cover for tall herbaceous vegetation. These conditions are favorable for many *Orthoptera* species, which are able to find refuge from predators in tall grass (Batáry et al., 2007; Gavlas et al., 2007; Sutcliffe et al., 2015). This confounding effect between herbicide use and vegetation structure and humidity made it difficult to detect a possible herbicide effect on these insects, which could be balanced by other favorable conditions. Furthermore, some studies have already shown that *Orthoptera* can be highly resistant to pesticides (Brahimi et al., 2020).

### 4.2 Connectivity effects

All taxa studied seemed to be influenced by site connectivity as well. Thus, the flora was more abundant on the best‐connected sites, but we did not observe any significant relationship between plant richness and current site connectivity. Similar to Lindborg and Eriksson (2004), the current plant diversity we observed is probably better explained by the past landscape than by the current one, or else we poorly evaluated the resistance costs of land uses when modeling.

The abundance of *Rhopalocera* was most strongly linked to site connectivity. Several previous studies in highly fragmented contexts have shown a positive effect of grassland connectivity on butterflies (Brückmann et al., 2010; Pöyry et al., 2009). Studies showing little or no effects on butterfly abundance were conducted in areas with a large amount of favorable habitat (Villemey et al., 2015). Our results therefore highlight the potential effect of landscape fragmentation in the study area and suggest that *Rhopalocera* species could be limited by the low connectivity of open habitats.

Other studies conducted on the influence of connectivity on *Orthoptera* have shown an absence of effects (Löffler & Fartmann, 2017) or a positive effect (Badenhausser & Cordeau, 2012). We observed higher evenness on the most connected sites. We suppose that highly mobile species maintain many local metapopulations with high turnover rates, reducing the likelihood that a few species will become numerically dominant.

### 4.3 Local effects

The richness and abundance of flora on the sites seemed to depend mainly on the soil type. Sandy soils, which are poorer, may favor greater diversity of species than clay soils, which are richer and where competition could reduce species richness due to interspecific competitive exclusion (Rajaniemi, 2002). Conversely, the insect communities studied were globally more linked to the landscape than to local site conditions. This result is contrary to several studies showing an equivalent (Sutcliffe et al., 2015) or even greater (Pöyry et al., 2009) effect of local conditions than that of landscape variables. Nectar availability being a limiting factor for butterflies on the landscape scale (Franzén & Nilsson, 2008), we expected that floral availability would have a greater influence on *Rhopalocera* communities. However, we still observed that butterflies were more abundant when the plants were more diversified.

This was not the case for *Orthoptera*, whose richness decreased with increased plant diversity. Indeed, *Orthoptera* species are known to react more to different vegetation strata than to the diversity of plant species (e.g., Fartmann et al., 2012). However, the low vegetation cover did not seem to influence abundance, diversity, and evenness of those insects. It should be remembered that site moisture and the high herbaceous stratum could not be considered in the analysis because these variables were too dependent on pesticide use, but probably influenced the results in our case. The contradictory correlations between the diversity measures we obtained for *Orthoptera* and *Rhopalocera* could be explained by the differences in the plant species on which they depend.

### 4.4 Functional traits

We expected that specialist and less mobile species would suffer more from habitat isolation (Keller et al., 2013; Miller et al., 2015; Villemey et al., 2015). Our results showed that site connectivity could favor low‐dispersal plants, while herbicide use had a greater negative impact on specialist and less mobile plants. However, the dispersal capacities of the target species of the herbicides used in the study area were not significantly lower. It was mainly competitive and highly dispersive tall grasses that grew on sites around which herbicides were used. We can assume that they could have colonized sites to the detriment of more pesticide‐sensitive species, which are probably less mobile and less generalist. Furthermore, the vast majority of *Rhopalocera* and *Orthoptera* recorded were species with a high degree of dispersal and low specialization, probably selected in such a highly artificial and fragmented landscape (Rochat et al., 2017). Our results showed an increase in *Orthoptera* community specialization when the cover for low herbaceous vegetation increased. Indeed, the species considered in the study as specialists were dependent on xeric environments and could have been favored by lower vegetation. *Orthoptera* community specialization also tended to be higher on more connected sites, suggesting greater sensitivity of specialist species to the isolation of their habitat.

### 4.5 Scale‐dependent processes

Vascular‐plant and hexapod communities on industrial water‐abstraction sites were influenced by both local and landscape factors, with overall a greater effect of local factors on flora richness and abundance and a greater effect of landscape factors on these same measures for insects. The intensity of site management, which induces disturbance and changes in habitat, is often a local factor that has a strong influence on species communities in herbaceous environments (e.g., Stoner & Joern, 2004). In our case, management was the same on our sites, which may partly explain our results. Conversely, landscape factors mainly influenced the functional diversity of flora, and local factors mainly influenced that of *Orthoptera*. This study shows the importance of taking into account different dimensions of biodiversity and different spatial scales to better understand ecological processes.

## 5 CONCLUSION

Similar to other types of industrial sites, water‐abstraction sites, when managed ecologically, can constitute seminatural habitats in landscapes that are increasingly fragmented by agricultural intensification and urbanization. They could play a key role in biodiversity by providing habitats and refuges for species, and by improving landscape connectivity. A landscape‐wide approach involving local stakeholders would be more effective in conserving and restoring biodiversity, given the influence of the landscape on the species found on the sites. Partnerships and discussions with farmers should be favored and pursued because the impact of their practices was preponderant in our results.

## CONFLICT OF INTEREST

The authors have declared no conflict of interest.

## AUTHOR CONTRIBUTION

**Chloé Thierry:** Data curation (lead); Formal analysis (supporting); Methodology (lead); Writing‐original draft (lead). **Benoît Pisanu:** Formal analysis (lead); Writing‐review & editing (supporting). **Nathalie Machon:** Supervision (lead); Writing‐review & editing (supporting).

## Supporting information

Supplementary Material

## Data Availability

The data used in this article are available in the "supplementary material" document. They are also archived in Zenodo: https://doi.org/10.5281/zenodo.5572697.
