# Supplementary material for: Both landscape and local factors influence plant and hexapod communities of industrial water‐abstraction sites
Source: Ecol Evol. 2022 Feb 17;12(2):e8365. doi: 10.1002/ece3.8365 (PMC8855018; doi:10.1002/ece3.8365)
Supplement: Supplementary file 1 — Supplementary Material [file ECE3-12-e8365-s001.pdf]

# Both landscape and local factors influence plant and hexapod communities of industrial water-abstraction sites

Thierry C, Pisanu B, Machon N

August, 13th, 2021

Contents

1. Study sites and land-use categories for connectivity metrics **5**
1.1. Study sites . . . 5
1.2. Land-use categories . . . 6
1.3. Map used to infer connectivity index for *Rhopalocera* . . . 7

2. Data management and analyses for vascular flora **8**
2.1 Renaming columns, calculating Evenness, scaling explanatory variables . . . 8
2.2 Colinearity . . . 11
2.3. Data analysis . . . 12
2.3.1. Linear models for total vascular plant species . . . 12
2.3.1.1 Species richness . . . 12
a. Variance checking . . . 12
b. Model coefficients . . . 12
2.3.1.2 Total abundance . . . 14
a. Variance checking . . . 14
b. Model coefficients . . . 14
2.3.1.3 Evenness . . . 16
a. Variance checking . . . 16
b. Model coefficients . . . 16
2.3.1.4 Community dispersion . . . 18
a. Variance checking . . . 18
b. Model coefficients . . . 18
2.3.1.5 Community specialisation . . . 19
a. Variance checking . . . 19
b. Model coefficients . . . 19

2.3.1.6 Community pollinisation 20
a. Variance checking 20
b. Model coefficients 20
2.3.2. Figure for plant analyses (Fig. 3 of the ms) 21

3. Data management and analyses on Rhopaloceran species **22**
3.1. Renaming columns, calculating Evenness, scaling expalantory variables 22
3.2. Colinearity 24
3.3. Data analysis 25
3.3.1. Species richness 25
a. Variance checking 25
b. Model coefficients 25
3.3.2. Abundance 26
a. Variance checking 26
b. Model coefficients 27
3.3.3. Evenness 28
a. Variance checking 28
b. Model coefficients 28
3.3.4. Community dispersion metric 29
a. Variance checking 29
b. Model coefficients 30
3.3.5. Community specialisation metric 31
a. Variance checking 31
b. Model coefficients 31
3.4. Figure (Fig. 4 of the ms) 32

4. Data management and analyses for Orthopteran species **33**
4.1 Renaming columns, calculating Evenness, scaling explanatory variables 33
4.2 Colinearity 36
4.3 Data analysis 37
4.3.1. Species richness 37
a. Variance checking 37
b. Model coefficients 37
4.3.2. Abundance 38
a. Variance checking 38
b. Model coefficients 39
4.3.3. Evenness 40

a. Variance checking 40

b. Model coefficients 40

4.3.4. Community specialisation 41

a. Variance checking 41

b. Model coefficients 42

4.4. Figure (Fig. 5 of the ms) 43

5. Correlations between community diversity measures (Table 4 of the ms) **44**

Packages and versions used

```
# Analyses run in Rstudio v.1.4.1717 with R 4.1.0.

#  R Core Team (2021). R: A language and environment for statistical computing.
# R Foundation for Statistical Computing, Vienna, Austria.
# URL https://www.R-project.org/.

# RStudio Team (2021). RStudio: Integrated Development Environment for R.
# RStudio, PBC, Boston, MA URL http://www.rstudio.com/.)

library(MASS) # 7.3-54
library(plyr) # 1.8.6
library(dplyr) # 1.0.6
library(sp) # 1.4-5
library(maps) # 3.3.0
library(osmar) # 1.1-7
library(ggplot2) # 3.3.5
library(ggmap) # 3.0.0
library(ggsn) # 0.5.0
source(file = "HighstatLibV6.R") # Zuur et al 2013 BGGLMMwR -
# Highland Statistics Inc http://www.highstat.com/BGGLM.htm
library(ncf) # 1.2-9
library(gridExtra) # 2.3
library(scales) # 1.1.1
library(kableExtra) #1.3.4
library(png) #0.1-7
library(knitr) #1.33
```

1. Study sites and land-use categories for connectivity metrics

1.1. Study sites

```
Data_flora<-read.table("GLM3_Flore_global_vfd.txt", h=T)
Data_flora$Y<-as.numeric(Data_flora$Y)
xy_Data_flora<-data.frame(x=Data_flora$X, y=Data_flora$Y)
coordinates(xy_Data_flora)<-c("x", "y")
proj4string(xy_Data_flora) <- CRS("+init=epsg:2154")
CRS.new <- CRS("+proj=longlat +ellps=WGS84 +datum=WGS84 +no_defs")

newcoordWS <- spTransform(xy_Data_flora, CRS.new)
Data_flora$x_WGS<-newcoordWS$x
Data_flora$y_WGS<-newcoordWS$y

map_Water_sites <- get_map(location = c(left = 1.7, bottom = 48.9,
                                right = 1.9,top = 49),
                   maptype =c("terrain"), source = "osm")
Water_site_2020_map<-ggmap(map_Water_sites)+
  theme(legend.position = "bottom", legend.title = element_blank(),
        plot.margin = margin(0.1,0.1,0.1,0.1, "cm"))+
  geom_point(data = Data_flora, aes(x = x_WGS, y = y_WGS), pch=20, col="red",size=3)+
  labs(y = "Latitude (N, WGS84)")+
  labs(x = "Longitude (E, WGS84)")+
  scalebar(x.min=1.7, x.max=1.9, y.min=48.9,  y.max=49,
           dist = 1, transform = TRUE, dist_unit = "km", model = 'WGS84',
            box.fill = c("black", "white"), st.color = "black")+
    geom_text(x=1.875, y=48.905, label="0", size=2.5)+
  geom_text(x=1.888, y=48.905, label="1 km", size=2.5)+
  labs(caption = "Figure 1.1: Location of study sites")+
  theme(plot.caption = element_text(hjust=0.5, size=rel(1.1)))

Water_site_2020_map
```

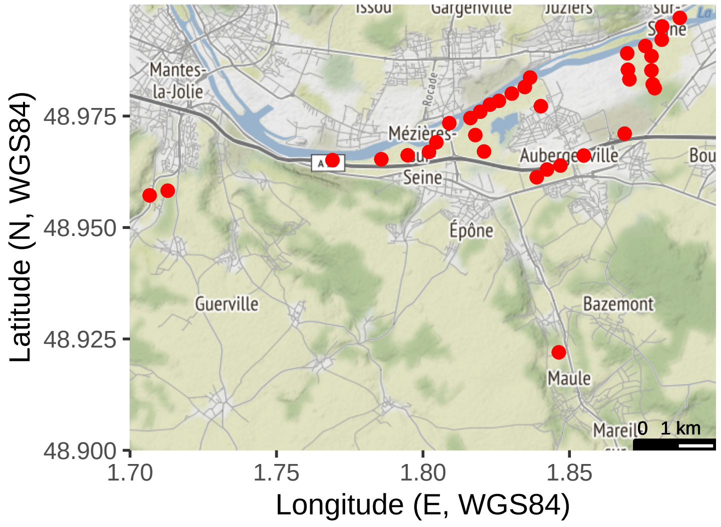

Figure 1.1: Location of study sites

1.2. Land-use categories

```
Land_use_levels<-read.table("land_use_categories.txt", h=T)
Land_use_levels  %>%
kable(caption =
      "Table 1.2. Costs allocated to each land-use category,
      according to its resistance to movement for each taxon") %>%
kable_styling(bootstrap_options = c("striped", "hover"), font_size = 8,
              latex_options = "HOLD_position") %>%
row_spec(0,bold=TRUE)
```

Table 1.2. Costs allocated to each land-use category, according to its resistance to movement for each taxon

**Land_use_categories**	**Flora**	**Rhopalocera**	**Orthoptera**
Semi_natural_or_natural_herbaceous_environments	1	1	1
Artificial_herbaceous_environments	1	10	10
Herbaceous_&_shrubby_environments	10	10	10
Herbaceous_&_wooded_environments	10	50	50
Shrubby_environments	10	100	100
Shrubby_&_herbaceous_environments	10	50	50
Wooded_environments	1000	1000	1000
Wooded_&_herbaceous_environments	10	100	100
Crops	10	100	100
Areas_without_vegetation	10	100	10
Water_surfaces	10	100	100
Artificial_areas	10	100	100
Buildings_up_to_2_m	10	100	100
Buildings_from_3_to_10_m	10	100	1000
Buildings_from_11_to_15_m	10	1000	1000
Buildings_from_16_m	1000	1000	1000
Primary_roads	10	1000	1000
Secondary_roads	10	100	100

1.3. Map used to infer connectivity index for *Rhopalocera*

```
img1_path <- "Thierry_et_al_Fig_SM1_3.png"
include_graphics(img1_path)
```

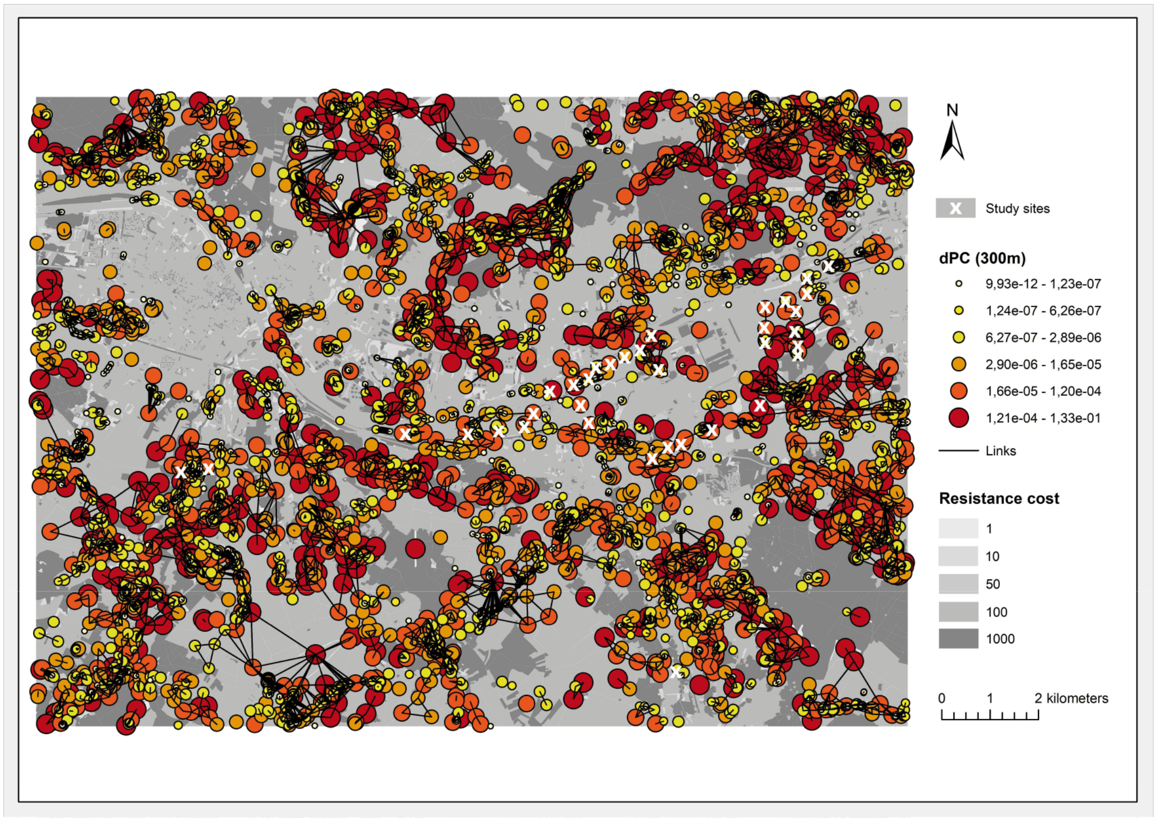

2. Data management and analyses for vascular flora

2.1 Renaming columns, calculating Evenness, scaling explanatory variables

```
Data_flora<-Data_flora[,c("Site", "X", "Y", "Richesse", "Richesse_pour_evenness",
                        "Diversite_Shannon", "Abondance", "CWM_dispersion", "CWM_specialisation",
                        "CWM_dep_pol", "dPC_Flore_500m", "IFT_HERB_min_NA2_300m",
                        "dPC_Rhopaloceres_300m", "Sol","Humidite_num")]
# Identifying columns
colnames(Data_flora) <-c("Site"="Site",                                             #1
                         "X"="X",                                                   #2
                         "Y"="Y",                                                   #3
                         "Richesse"="RSp",                                          #4
                         "Richesse_pour_evenness"="RSpE",                           #5
                         "Diversite_Shannon"="Htot",                                #6
                         "Abondance"="Abtot",                                       #7
                         "CWM_dispersion"="CWMd",                                   #8
                         "CWM_specialisation"="CWMs",                               #9
                         "CWM_dep_pol"="CWMdp",                                     #10
                         "dPC_Flore_500m"="dPCF500",                                #11
                         "IFT_HERB_min_NA2_300m"="IFTher300",                       #12
                         "dPC_Rhopaloceres_300m"="dpcR300",                         #13
                         "Sol"="Soil",                                              #14
                         "Humidite_num")                                            #15


Data_flora$Soil<-revalue(Data_flora$Soil, c("Argileux"="Clay", "Sableux"="Sandy"))
Data_flora$Soil2<-as.numeric(revalue(Data_flora$Soil, c("Clay"=1, "Sandy"=2)))


# Pielou Evenness - E=H/log2(S)
Data_flora$EFtot<-Data_flora$Htot/log2(Data_flora$RSpE)

# Scaling variables
Data_flora$dPCF500<-scale(Data_flora$dPCF500)
Data_flora$dpcR300<-scale(Data_flora$dpcR300)
Data_flora$IFTher300<-scale(Data_flora$IFTher300)
```

```r
print(Data_flora)
```

```
##    Site        X       Y RSp RSpE     Htot Abtot      CWMd     CWMs    CWMdp
## 1    A1 614844.9 6876594  26   26 3.048667   130 1.0385385 17.18266 57.38710
## 2   A10 611759.1 6874702  21   20 2.818922    97 1.2652747 17.60140 51.05495
## 3   A11 611097.9 6874614  13   11 2.186421    70 1.2305263 19.03911 34.41818
## 4   A12 613667.2 6874767  28   26 3.036171   131 0.9990698 17.72780 50.92248
## 5   A13 609876.1 6874610  10   10 2.038113    47 1.3442553 18.14083 42.00000
## 6   A14 612479.5 6875016  14   12 2.279555    84 1.2486486 19.77419 28.72222
## 7   A15 613456.2 6875181   8    8 1.731350    34 1.2261765 18.87681 29.17647
## 8    A2 614709.6 6876360  18   15 2.531624   109 1.2552632 18.58878 41.50000
## 9    A3 614056.8 6876024  14   14 2.413525    81 1.0470370 18.63649 40.55000
## 10   A4 613831.3 6875933  26   26 3.031157   135 0.9917164 17.82707 53.21481
## 11   A5 613340.1 6875607  22   22 2.814181   101 0.8351485 17.21313 47.11881
## 12   A6 614382.3 6876201  18   17 2.581524    86 1.1466667 17.78569 55.56410
## 13   A7 613586.1 6875760  11   10 2.102132    67 0.9400000 18.68224 48.96923
## 14   A8 612810.2 6875487  13   13 2.489472    92 0.8756522 17.36072 37.20652
## 15   A9 612291.7 6874778  12   12 2.192885    60 0.9896667 18.97475 27.36667
## 16  C10 617280.6 6877163  21   19 2.666111    96 1.0759783 18.67776 43.88043
## 17  C11 618606.9 6878027  13   12 2.327956    79 0.8967105 17.11607 51.82857
## 18  C12 618163.2 6877820  14   13 2.394347    83 0.8756098 18.74913 46.76923
## 19   C2 617954.0 6876284  29   28 3.204026   191 0.4773936 17.81353 44.10526
## 20   C3 617907.2 6876382  28   27 3.015726   174 0.5617647 18.17829 43.04094
## 21   C4 617879.8 6876726  35   32 3.261601   180 0.7312048 17.39880 54.11377
## 22   C5 617326.8 6876514  29   26 3.072174   136 0.8419828 18.82222 47.70248
## 23   C6 617292.0 6876753  17   16 2.542829    92 0.8941772 17.36214 58.29114
## 24   C7 617886.3 6877084  19   17 2.726213   110 0.9791919 17.92448 49.03030
## 25   C8 618147.5 6877491  26   25 2.957280   128 1.0313386 17.86489 53.41270
## 26   C9 617736.0 6877338  17   15 2.581664   121 1.0921818 18.01449 43.74545
## 27   LF 615104.1 6875877  26   24 2.898878   112 1.1377778 17.34791 49.28713
## 28   MF 615462.3 6869730  22   18 2.685232    97 0.9374725 17.95912 55.51111
## 29   P1 617188.1 6875161  30   28 3.199327   203 0.4213441 18.94834 52.67347
## 30   P2 616158.3 6874631  23   22 2.934159   160 0.6823448 18.21086 49.88667
## 31   P3 615571.0 6874389  31   29 3.206545   193 0.6296216 18.43825 51.11351
## 32   P4 614974.6 6874105  22   22 2.842377   109 0.9000971 18.85358 49.78241
## 33   P5 615233.6 6874295  23   21 2.883032   144 0.7517730 18.03600 62.70423
## 34   V2 605292.9 6873804  16   15 2.477129    87 1.1545455 18.08463 28.06494
## 35  V36 605746.3 6873914  24   23 2.924335   111 1.3654545 17.57257 44.11818
##       dPCF500   IFTher300      dpcR300  Soil Humidite_num Soil2     EFtot
## 1  -0.4152318 -0.18391264 -0.228306633 Sandy            3     2 0.6485919
## 2  -0.4301196  1.36509243 -0.229620929  Clay            3     1 0.6522372
## 3  -0.3420702  0.65404236 -0.203338973 Sandy            3     2 0.6320175
## 4  -0.1119854  1.56759542 -0.171683139 Sandy            3     2 0.6459333
## 5  -0.3977237 -0.51577005 -0.171542664  Clay            3     1 0.6135332
## 6  -0.4054370  1.83845716 -0.216071568 Sandy            2     2 0.6358659
## 7  -0.3857006  2.79949316 -0.197118043  Clay            3     1 0.5771166
## 8  -0.1692067  0.44076224  0.124757440 Sandy            3     2 0.6479895
## 9  -0.4319687  0.45599130 -0.229200469 Sandy            3     2 0.6339113
## 10 -0.4197939  0.01750980 -0.227523159 Sandy            3     2 0.6448666
## 11 -0.3230601  0.27345724 -0.007926949 Sandy            3     2 0.6310626
## 12 -0.4363001  0.79006983 -0.232794945  Clay            3     1 0.6315714
## 13 -0.4069309  0.05377458 -0.214984918  Clay            3     1 0.6328047
## 14 -0.2447052 -0.26776687  0.149674046  Clay            3     1 0.6727504
```

```
## 15 -0.4294348  2.19228367 -0.231415908  Clay             3     1 0.6116899
## 16 -0.2629402 -0.82465436 -0.216547248  Clay             2     1 0.6276262
## 17 -0.4356750 -0.18398015 -0.226994206  Clay             3     1 0.6493669
## 18 -0.3981141 -0.60663101 -0.234895055  Clay             3     1 0.6470439
## 19 -0.4014612 -0.82465436 -0.211887472 Sandy            1     2 0.6664842
## 20 -0.3799747 -0.82465436 -0.174711927 Sandy            1     2 0.6342371
## 21  2.9241183 -0.82465436  5.702300322 Sandy            2     2 0.6523201
## 22 -0.2268387 -0.82465436  0.315551544 Sandy            2     2 0.6535928
## 23 -0.2629402 -0.82465436 -0.207182444 Sandy            2     2 0.6357073
## 24 -0.4040965 -0.82465436 -0.222210219 Sandy            2     2 0.6669694
## 25 -0.4335939 -0.82000533 -0.224634730  Clay             2     1 0.6368155
## 26 -0.4095871 -0.82465436 -0.220489590  Clay             3     1 0.6607976
## 27 -0.4342002 -0.80387916 -0.239316055 Sandy            2     2 0.6322577
## 28 -0.4350559          NA -0.243650440  Clay             3     1 0.6439520
## 29  3.9526609 -0.82465436 -0.091820348 Sandy            1     2 0.6655068
## 30  2.2574213 -0.82465436 -0.194855820 Sandy            2     2 0.6579671
## 31  0.4943367 -0.20895673 -0.190027721 Sandy            2     2 0.6600572
## 32  0.4943367 -0.28468670 -0.228504172 Sandy            2     2 0.6373855
## 33 -0.3509565 -0.32639695 -0.222868995 Sandy            2     2 0.6563807
## 34  0.4319800          NA -0.147793803  Clay             2     1 0.6340411
## 35 -0.3697510          NA -0.232364806  Clay             2     1 0.6464674
```

2.2 Colinearity

```
Flora_plot1<-pairs(Data_flora[c(11:13,15:16)],
                upper.panel = panel.lines2, lower.panel=panel.cor, digits=2)
```

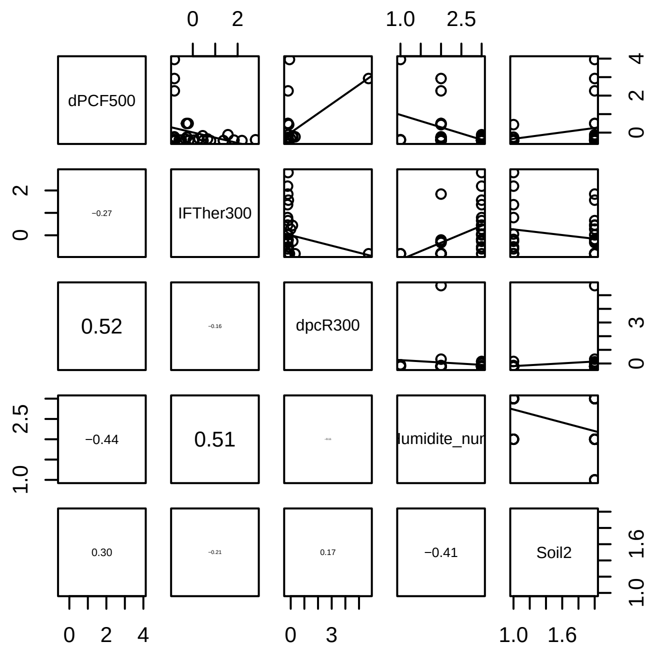


```
pourVIF_Es1<-Data_flora[c(11:13,16)]
corvif(pourVIF_Es1)
```

```
##
##
## Variance inflation factors
##
##                 GVIF
## dPCF500   1.521362
## IFTher300 1.102255
## dpcR300   1.367823
## Soil2     1.122013
```

We confirm weak collinearity was detected (correlation coefficients between variables less than *0.30*; GVIFs $<$ 2) when only including connectivity indice (radius 500m; *dPCF500*), level or intensity of herbicide treatments (radius 300m; *IFTher300*), and soil types (*Soil*) as explanatory variables in the statistical analyses. Relative humidity was not retained in the stastitical analyses as it correlates above r = *0.30* with all other explanatory variables.

2.3. Data analysis

2.3.1. Linear models for total vascular plant species

2.3.1.1 Species richness

```
mod_Rsp <- lm(RSp ~ dPCF500+IFTher300+Soil,
            data = Data_flora)

par(mfrow=c(1,3))
plot(mod_Rsp, which=1:2, caption = list("Gaussian", "QQ_plot","", "",""), cex.caption = 0.8)
spatcor_Es<-spline.correlog(x=Data_flora[, "X"], y=Data_flora[, "Y"],
z=residuals(mod_Rsp, type="pearson"),na.rm=TRUE, xmax=3000,
resamp=100, npoints=30, quiet = T)
plot(spatcor_Es)
```

a. Variance checking

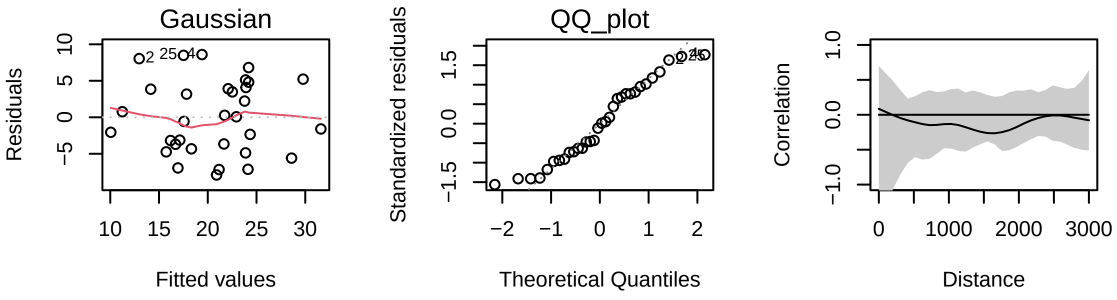

The linear model provides a good fit to the data. No spatial autocorrelation is detected.

```
round(summary(mod_Rsp)$coefficients,3)
```

b. Model coefficients

```
##              Estimate Std. Error t value Pr(>|t|)
## (Intercept)    16.572      1.575  10.524    0.000
## dPCF500         1.773      0.981   1.808    0.081
## IFTher300      -2.084      0.993  -2.100    0.045
## SoilSandy       6.302      2.037   3.094    0.004
```

```
Fl_RSp<-ddply(Data_flora, .(Soil), summarize,
n=length(RSp),
mean = round(mean(RSp),2),
se = round(sd(RSp)/sqrt(n), 2))
Fl_RSp
```

```
##    Soil  n  mean   se
## 1  Clay 15 16.40 1.41
## 2 Sandy 20 23.65 1.39
```

```r
mod_Ab <- lm(Abtot ~ dPCF500+IFTher300+Soil,
             data = Data_flora)
```

2.3.1.2 Total abundance

```r
par(mfrow=c(1,3))
plot(mod_Ab, which=1:2, caption = list("Gaussian", "QQ_plot","", "",""), cex.caption = 0.8)
spatcor_Es<-spline.correlog(x=Data_flora[, "X"], y=Data_flora[, "Y"],
z=residuals(mod_Ab, type="pearson"),na.rm=TRUE, xmax=3000,
resamp=100, npoints=30, quiet = T)
plot(spatcor_Es)
```

a. **Variance checking**

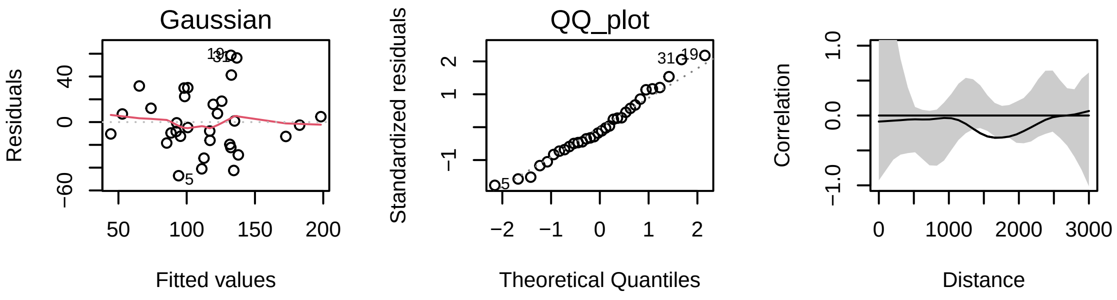

The linear model provides a good fit to the data. No spatial autocorrelation is detected.

```r
round(summary(mod_Ab)$coefficients,3)
```

b. **Model coefficients**

```
##               Estimate Std. Error t value Pr(>|t|)
## (Intercept)     92.345      8.415  10.974    0.000
## dPCF500         15.129      5.241   2.887    0.007
## IFTher300      -15.035      5.305  -2.834    0.008
## SoilSandy       33.717     10.884   3.098    0.004
```

```r
Fl_Ab<-ddply(Data_flora, .(Soil), summarize,
n=length(Abtot),
mean = round(mean(Abtot),2),
se = round(sd(Abtot)/sqrt(n), 2))
Fl_Ab
```

```
##    Soil  n   mean   se
## 1  Clay 15  85.67 6.66
## 2 Sandy 20 132.25 8.96
```

```
mod_EFtot <- lm(EFtot ~ dPCF500+IFTher300+Soil,
            data = Data_flora)
```

2.3.1.3 Evenness

```
par(mfrow=c(1,3))
plot(mod_EFtot, which=1:2, caption = list("Gaussian", "QQ_plot","", "",""), cex.caption = 0.8)
spatcor_Es<-spline.correlog(x=Data_flora[, "X"], y=Data_flora[, "Y"],
z=residuals(mod_EFtot, type="pearson"),na.rm=TRUE, xmax=3000,
resamp=100, npoints=30, quiet = T)
plot(spatcor_Es)
```

a. Variance checking

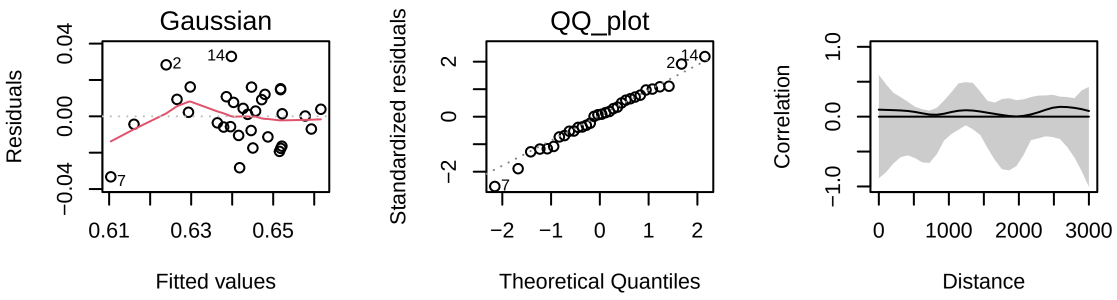

The linear model provides a good fit to the data. No spatial autocorrelation is detected.

```
round(summary(mod_EFtot)$coefficients,3)
```

b. Model coefficients

```
##             Estimate Std. Error t value Pr(>|t|)
## (Intercept)    0.638      0.005 134.847    0.000
## dPCF500        0.002      0.003   0.765    0.451
## IFTher300     -0.009      0.003  -3.180    0.004
## SoilSandy      0.007      0.006   1.156    0.257
```

```
Fl_E<-ddply(Data_flora, .(Soil), summarize,
n=length(EFtot),
mean = round(mean(EFtot),3),
se = round(sd(EFtot)/sqrt(n), 3))
Fl_E
```

```
##    Soil  n  mean    se
## 1  Clay 15 0.636 0.006
## 2 Sandy 20 0.647 0.003
```

```
mod_CWMd <- lm(CWMd ~ dPCF500+IFTher300+Soil,
             data = Data_flora)
```

2.3.1.4 Community dispersion

```
par(mfrow=c(1,3))
plot(mod_CWMd, which=1:2, caption = list("Gaussian", "QQ_plot","", "",""), cex.caption = 0.8)
spatcor_Es<-spline.correlog(x=Data_flora[, "X"], y=Data_flora[, "Y"],
z=residuals(mod_CWMd, type="pearson"),na.rm=TRUE, xmax=3000,
resamp=100, npoints=30, quiet = T)
plot(spatcor_Es)
```

a. Variance checking

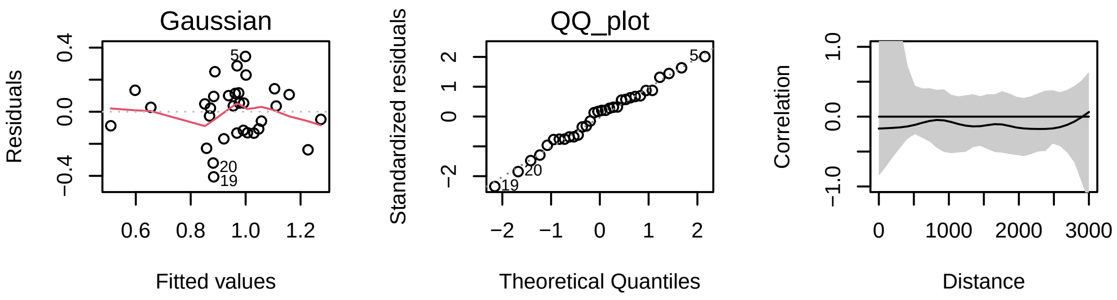

The linear model provides a good fit to the data. No spatial autocorrelation is detected.

```
round(summary(mod_CWMd)$coefficients,3)
```

b. Model coefficients

```
##             Estimate Std. Error t value Pr(>|t|)
## (Intercept)    1.008      0.054  18.596    0.000
## dPCF500       -0.086      0.034  -2.549    0.017
## IFTher300      0.083      0.034   2.433    0.022
## SoilSandy     -0.090      0.070  -1.289    0.208
```

```
mod_CWMs <- lm(CWMs ~ dPCF500+IFTher300+Soil,
            data = Data_flora)
```

2.3.1.5 Community specialisation

```
par(mfrow=c(1,3))
plot(mod_CWMs, which=1:2, caption = list("Gaussian", "QQ_plot","", "",""), cex.caption = 0.8)
spatcor_Es<-spline.correlog(x=Data_flora[, "X"], y=Data_flora[, "Y"],
z=residuals(mod_CWMs, type="pearson"),na.rm=TRUE, xmax=3000,
resamp=100, npoints=30, quiet = T)
plot(spatcor_Es)
```

a. Variance checking

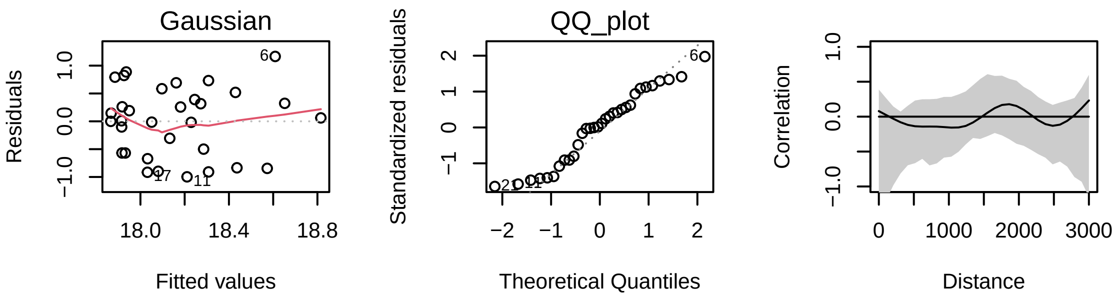

The linear model provides a good fit to the data. No spatial autocorrelation is detected.

```
round(summary(mod_CWMs)$coefficients,3)
```

b. Model coefficients

```
##             Estimate Std. Error t value Pr(>|t|)
## (Intercept)   18.131      0.195  92.781    0.000
## dPCF500        0.118      0.122   0.971    0.340
## IFTher300      0.261      0.123   2.117    0.043
## SoilSandy      0.046      0.253   0.184    0.856
```

```
mod_CWMdp <- lm(CWMdp ~ dpcR300+IFTher300+Soil,
            data = Data_flora)
```

2.3.1.6 Community pollinisation

```
par(mfrow=c(1,3))
plot(mod_CWMdp, which=1:2, caption = list("Gaussian", "QQ_plot","", "",""), cex.caption = 0.8)
spatcor_Es<-spline.correlog(x=Data_flora[, "X"], y=Data_flora[, "Y"],
z=residuals(mod_CWMdp, type="pearson"),na.rm=TRUE, xmax=3000,
resamp=100, npoints=30, quiet = T)
plot(spatcor_Es)
```

a. Variance checking

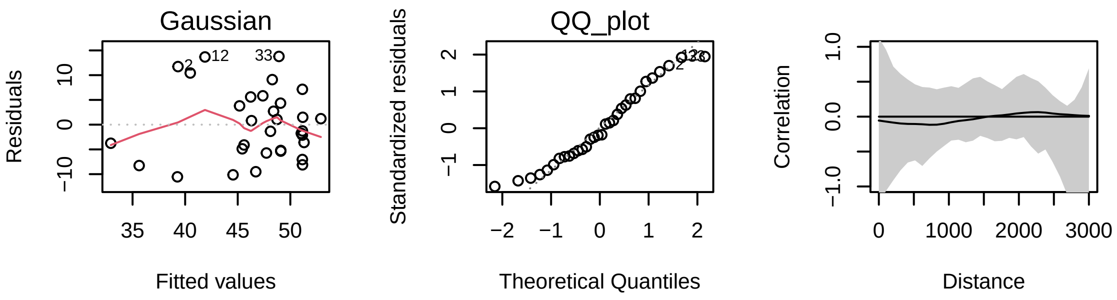

The linear model provides a good fit to the data. No spatial autocorrelation is detected.

```
round(summary(mod_CWMdp)$coefficients,3)
```

b. Model coefficients

```
##             Estimate Std. Error t value Pr(>|t|)
## (Intercept)   45.477      2.168  20.974    0.000
## dpcR300        0.297      1.296   0.229    0.820
## IFTher300     -4.461      1.367  -3.263    0.003
## SoilSandy      2.054      2.776   0.740    0.466
```

2.3.2. Figure for plant analyses (Fig. 3 of the ms)

```
Fig10<-ggplot(Data_flora, aes(x=IFTher300, y=RSp)) +
  scale_y_continuous(labels = function(x) round(as.numeric(x), digits=2)) +
  geom_point() + stat_smooth(method="lm", se=T) +
  ylab("Species richness") + xlab("")+theme_classic(base_size = 11)+
  theme(plot.margin=unit(c(0.5,0.5,0,0.5), "cm"))
Fig1a<-ggplot(Data_flora, aes(x=IFTher300, y=EFtot)) +
  scale_y_continuous(labels = function(x) round(as.numeric(x), digits=2)) +
  geom_point() + stat_smooth(method="lm", se=T) +
  ylab("Evenness") + xlab("")+theme_classic(base_size = 11)+
  theme(plot.margin=unit(c(0.5,0.5,0,0.5), "cm"))

Fig_x1<-grid.arrange(Fig10,Fig1a,nrow = 1, ncol = 2,
bottom = textGrob("Rate of herbicide treatment within 300 m",
rot = 0, vjust = 0.1, hjust=0.4,gp=gpar(fontsize=11)))
```

```
Fig1b<-ggplot(Data_flora, aes(x=IFTher300, y=CWMd)) +
  scale_y_continuous(labels = function(x) round(as.numeric(x), digits=2)) +
  geom_point() + stat_smooth(method="lm", se=T) +
  ylab("Dispersal") + xlab("")+ theme_classic(base_size = 11)+
  theme(plot.margin=unit(c(0.5,0.5,0,0.5), "cm"))
Fig1c<-ggplot(Data_flora, aes(x=IFTher300, y=CWMs)) +
  scale_y_continuous(labels = function(x) round(as.numeric(x), digits=2)) +
  geom_point() + stat_smooth(method="lm", se=T) +
  ylab("Specialisation") + xlab("")+ theme_classic(base_size = 11)+
  theme(plot.margin=unit(c(0.5,0.5,0,0.5), "cm"))

Fig_x2<-grid.arrange(Fig1b,Fig1c,nrow = 1, ncol = 2,
bottom = textGrob("Rate of herbicide treatment within 300 m",
rot = 0, vjust = 0.1, hjust=0.4,gp=gpar(fontsize=11)))
```

```
Fig1d<-ggplot(Data_flora, aes(x=IFTher300, y=CWMdp)) +
  scale_y_continuous(labels = function(x) round(as.numeric(x), digits=2)) +
  geom_point() + stat_smooth(method="lm", se=T) +
  ylab("Pollinisation") + xlab("Rate of herbicide treatment within 300 m")+
  theme_classic(base_size = 11)+
  theme(plot.margin=unit(c(0.5,0.5,0,0.5), "cm"))
Fig1e<-ggplot(Data_flora, aes(x=dPCF500, y=Abtot)) +
  geom_point() + stat_smooth(method="lm", se=T) +
  ylab("Abundance") + xlab("Connectivity index (500m)")+
  theme(plot.title = element_text(size = 10, face = "bold"))+
  theme_classic(base_size = 11)+theme(plot.margin=unit(c(1,0.5,0,0.5), "cm"))
Fig_x3<-grid.arrange(Fig1d,Fig1e,nrow = 1, ncol = 2,
                     bottom = textGrob("",rot = 0, vjust = 0.1,
                                       hjust=0.4,gp=gpar(fontsize=11)))
```

```
Fig_1_0<-grid.arrange(Fig_x1,Fig_x2,Fig_x3, nrow = 3, ncol = 1)
```

```
Fig_1_0
#Figure 3 of the ms
#ggsave("Thierry_et_al_Fig_3.tiff", width = 16, height = 22, units="cm", dpi=300, Fig_1_0)
```

3. Data management and analyses on Rhopaloceran species

3.1. Renaming columns, calculating Evenness, scaling expalantory variables

```
Data_Rhopa<-read.table("GLM3_Rhopaloceres_global_vf.txt", h=T)
Data_Rhopa<-Data_Rhopa[,c("Site", "X", "Y", "Richesse", "Richesse_pour_evenness","Diversite_Shannon",
                          "Abondance", "CWM_dispersion", "CWM_specialisation", "dPC_Rhopaloceres_300m",
                          "IFT_HERB_min_NA2_300m", "Moy_dispo_florale", "Humidite_num")]

# renommer et numérote les colonnes pour simplifier la lecture des graphiques
colnames(Data_Rhopa) <-c( "Site"="Site",                        #1
                          "X"="X",                              #2
                          "Y"="Y",                              #3
                          "Richesse"="RSp",                     #4
                          "Richesse_pour_evenness"="RSpE",      #5
                          "Diversite_Shannon"="Htot",           #6
                          "Abondance"="Abtot",                  #7
                          "CWM_dispersion"="CWMd",              #8
                          "CWM_specialisation"="CWMs",          #9
                          "dPC_Rhopaloceres_300m"="dPCR300",    #10
                          "IFT_HERB_min_NA2_300m"="IFTher300", #11
                          "Moy_dispo_florale"="Flora",          #12
                          "Humidite_num"="Hum")                 #13

# calcul evenness de Pielou - E=H/log2(S)
Data_Rhopa$EFtot<-Data_Rhopa$Htot/log2(Data_Rhopa$RSpE)
# variables explicatives centrées réduites
Data_Rhopa$dPCR300<-scale(Data_Rhopa$dPCR300)
Data_Rhopa$IFTher300<-scale(Data_Rhopa$IFTher300)
Data_Rhopa$Flora<-scale(Data_Rhopa$Flora)
# sans les NA (3 valeurs manquantes)
Data_Rhopa<-Data_Rhopa[which(Data_Rhopa$IFTher300!="NA"),]
print(Data_Rhopa)
```

```
##    Site        X       Y RSp RSpE      Htot Abtot     CWMd     CWMs
## 1    A1 614844.9 6876594   8    8 1.8095480    24 2.625000 1.541667
## 2   A10 611759.1 6874702   4    3 1.0114043     8 2.750000 1.000000
## 3   A11 611097.9 6874614   1    1 0.0000000     1 2.000000 1.000000
## 4   A12 613667.2 6874767   9    9 1.9264330    23 2.913043 1.000000
## 5   A13 609876.1 6874610   5    5 1.3862944    10 2.900000 1.000000
## 6   A14 612479.5 6875016   3    2 0.6931472     3 2.666667 1.000000
## 7   A15 613456.2 6875181   9    9 1.6934100    26 2.730769 1.269231
## 8    A2 614709.6 6876360   5    4 1.3296613     6 2.666667 1.000000
## 9    A3 614056.8 6876024   4    4 1.1988493    12 2.750000 1.083333
## 10   A4 613831.3 6875933   1    1 0.0000000     1 2.000000 1.000000
## 11   A5 613340.1 6875607   7    7 1.6798181    26 2.730769 1.000000
## 12   A6 614382.3 6876201   5    5 1.5607104     9 3.000000 1.222222
## 13   A7 613586.1 6875760   4    4 1.3296613     6 3.000000 1.166667
## 14   A8 612810.2 6875487  10   10 2.0031636    26 2.880000 1.080000
## 15   A9 612291.7 6874778   4    3 1.0986123     8 2.000000 1.000000
## 16  C10 617280.6 6877163   8    8 1.7321260    16 2.933333 1.066667
## 17  C11 618606.9 6878027   8    8 2.0431919    11 2.818182 1.181818
```

```
## 18  C12 618163.2 6877820   7    7 1.8891592    21 3.000000 1.095238
## 19   C2 617954.0 6876284   8    7 1.7538124    31 2.516129 1.290323
## 20   C3 617907.2 6876382   7    7 1.5355669    36 2.611111 1.388889
## 21   C4 617879.8 6876726   6    6 1.6646580    86 2.694118 1.376471
## 22   C5 617326.8 6876514   9    9 1.6073568    69 2.808824 1.161765
## 23   C6 617292.0 6876753   6    6 1.5705321    68 2.805970 1.164179
## 24   C7 617886.3 6877084   7    7 1.6269052    75 2.840000 1.240000
## 25   C8 618147.5 6877491  10   10 1.9219056    34 2.735294 1.176471
## 26   C9 617736.0 6877338   7    6 1.6427799    40 2.846154 1.256410
## 27   LF 615104.1 6875877  12   12 2.3264185    28 2.750000 1.357143
## 29   P1 617188.1 6875161   4    4 1.3321790    10 2.800000 1.000000
## 30   P2 616158.3 6874631   5    4 1.0105254    47 2.913043 1.000000
## 31   P3 615571.0 6874389   6    6 1.1542932    32 2.937500 1.031250
## 32   P4 614974.6 6874105   6    6 1.2649232    54 2.962963 1.055556
## 33   P5 615233.6 6874295   7    7 1.5627106    27 2.960000 1.000000
##          dPCR300   IFTher300       Flora Hum     EFtot
## 1  -0.228306633 -0.18391264  1.00659851   3 0.6031827
## 2  -0.229620929  1.36509243  1.85896015   3 0.6381250
## 3  -0.203338973  0.65404236 -1.38001409   3       NaN
## 4  -0.171683139  1.56759542  0.35312125   3 0.6077220
## 5  -0.171542664 -0.51577005  0.40994536   3 0.5970445
## 6  -0.216071568  1.83845716 -1.29477792   2 0.6931472
## 7  -0.197118043  2.79949316 -0.61288861   3 0.5342114
## 8   0.124757440  0.44076224 -0.52765244   3 0.6648307
## 9  -0.229200469  0.45599130 -0.89700916   3 0.5994247
## 10 -0.227523159  0.01750980  0.55200563   3       NaN
## 11 -0.007926949  0.27345724 -1.12430559   3 0.5983633
## 12 -0.232794945  0.79006983 -0.30035601   3 0.6721614
## 13 -0.214984918  0.05377458 -0.78336094   3 0.6648307
## 14  0.149674046 -0.26776687 -0.78336094   3 0.6030123
## 15 -0.231415908  2.19228367 -1.20954176   3 0.6931472
## 16 -0.216547248 -0.82465436 -1.35160203   2 0.5773753
## 17 -0.226994206 -0.18398015  1.46119139   3 0.6810640
## 18 -0.234895055 -0.60663101  0.09741276   3 0.6729321
## 19 -0.211887472 -0.82465436  0.32470920   1 0.6247206
## 20 -0.174711927 -0.82465436 -0.55606450   1 0.5469800
## 21  5.702300322 -0.82465436  0.04058865   2 0.6439776
## 22  0.315551544 -0.82465436  0.60882974   2 0.5070646
## 23 -0.207182444 -0.82465436 -0.47082834   2 0.6075647
## 24 -0.222210219 -0.82465436  0.26788509   2 0.5795153
## 25 -0.224634730 -0.82000533 -0.84018505   2 0.5785512
## 26 -0.220489590 -0.82465436  0.52359358   3 0.6355140
## 27 -0.239316055 -0.80387916  1.23389495   2 0.6489380
## 29 -0.091820348 -0.82465436 -1.26636587   1 0.6660895
## 30 -0.194855820 -0.82465436  0.83612618   2 0.5052627
## 31 -0.190027721 -0.20895673  1.23389495   2 0.4465416
## 32 -0.228504172 -0.28468670  0.01217659   2 0.4893391
## 33 -0.222868995 -0.32639695  1.80213604   2 0.5566488
```

3.2. Colinearity

```
#correlogramme des variables explicatives
Rhopa_plot1<-pairs(Data_Rhopa[c(10:13)],
                upper.panel = panel.lines2, lower.panel=panel.cor, digits=2)
```

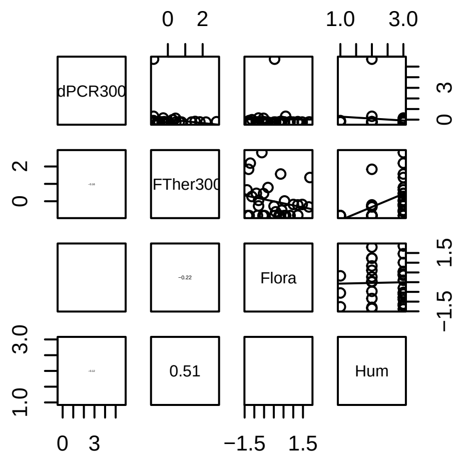


```
pourVIF_Es1<-Data_Rhopa[c(10:12)]
corvif(pourVIF_Es1)
```

```
##
##
## Variance inflation factors
##
##                GVIF
## dPCR300   1.029209
## IFTher300 1.081802
## Flora     1.053007
```

We confirm no collinearity was detected (correlations $<$ *0.30*; GVIFs $<$ 2) when including connectivity indice (radius 300m; *dPCR300*), level or intensity of herbicide treatments (radius 300m; *IFTher300*), and mean availability in flowering plant species (*Flora*) as explanatory variables in statistical analyses. Relative moisture was positively correlated (r = 0.51) to the rate of herbicide use and was not included in the analyses.

3.3. Data analysis

3.3.1. Species richness

```
mod_Rsp_Rhopa <- lm(RSp ~ dPCR300+IFTher300+Flora,
            data = Data_Rhopa)
```

```
par(mfrow=c(1,3))
plot(mod_Rsp_Rhopa, which=1:2, caption = list("Gaussian", "QQ_plot","", "",""), cex.caption = 0.8)
spatcor_Es<-spline.correlog(x=Data_Rhopa[, "X"], y=Data_Rhopa[, "Y"],
z=residuals(mod_Rsp_Rhopa, type="pearson"),na.rm=TRUE, xmax=3000,
resamp=100, npoints=30, quiet = T)
plot(spatcor_Es)
```

a. Variance checking

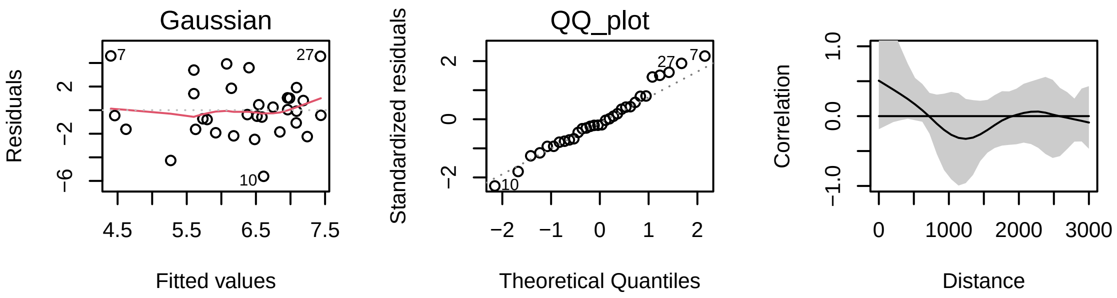

The linear model provides a good fit to the data. No spatial autocorrelation is detected.

```
round(summary(mod_Rsp_Rhopa)$coefficients,3)
```

b. Model coefficients

```
##             Estimate Std. Error t value Pr(>|t|)
## (Intercept)    6.326      0.443  14.287    0.000
## dPCR300       -0.079      0.437  -0.181    0.857
## IFTher300     -0.583      0.468  -1.247    0.223
## Flora          0.502      0.479   1.048    0.304
```

3.3.2. Abundance

```
mod_Ab_Rhopa0 <- lm(Abtot ~ dPCR300+IFTher300+Flora,
            data = Data_Rhopa)
mod_Ab_Rhopa <- lm(Abtot ~ dPCR300+IFTher300+Flora,
            data = Data_Rhopa,  subset=c(-7, -23,-24))
```

```
par(mfrow=c(2,3))
plot(mod_Ab_Rhopa0, which=1:2, caption = list("Gaussian", "QQ_plot","", "",""), cex.caption = 0.8)
spatcor_Es<-spline.correlog(x=Data_Rhopa[, "X"], y=Data_Rhopa[, "Y"],
z=residuals(mod_Ab_Rhopa0, type="pearson"),na.rm=TRUE, xmax=3000,
resamp=200, npoints=30, quiet = T)
plot(spatcor_Es)

plot(mod_Ab_Rhopa, which=1:2, caption = list("Gaussian", "QQ_plot","", "",""), cex.caption = 0.8)
Data_Rhopax<-Data_Rhopa[-c(7,23,24),]
spatcor_Esx<-spline.correlog(x=Data_Rhopax[, "X"], y=Data_Rhopax[, "Y"],
z=residuals(mod_Ab_Rhopa, type="pearson"),na.rm=TRUE, xmax=3000,
resamp=200, npoints=30, quiet = T)
plot(spatcor_Esx)
```

a. Variance checking

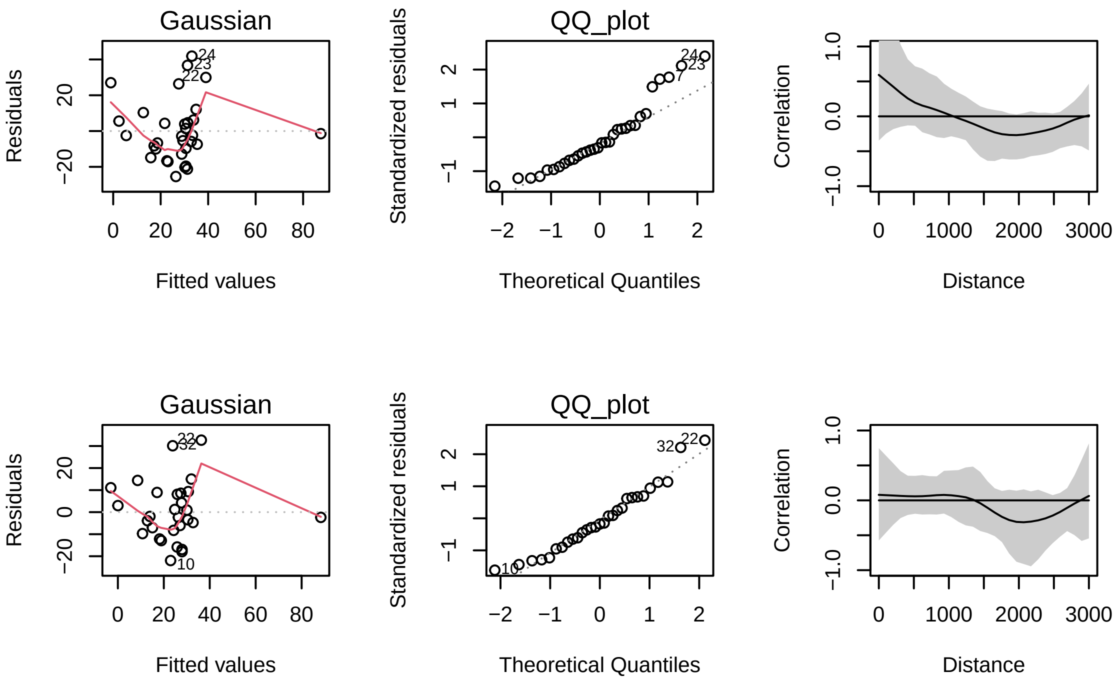

The linear model provides a relative poor fit to the data, due to 3 apparent outliers with excess in residual variance (7: site A15; 23: site C6; 24: site C7). Eliminating these outliers ameliorate variance homogeneity. No spatial autocorrelation is detected.

```
round(summary(mod_Ab_Rhopa0)$coefficients,3)
```

b. Model coefficients

```
##             Estimate Std. Error t value Pr(>|t|)
## (Intercept)   27.196      3.187   8.534    0.000
## dPCR300        9.272      3.142   2.951    0.006
## IFTher300     -8.838      3.366  -2.626    0.014
## Flora          2.671      3.448   0.775    0.445
```

```
round(summary(mod_Ab_Rhopa)$coefficients,3)
```

```
##             Estimate Std. Error t value Pr(>|t|)
## (Intercept)   23.509      2.586   9.089    0.000
## dPCR300       10.023      2.442   4.105    0.000
## IFTher300     -9.179      3.103  -2.958    0.007
## Flora          3.428      2.683   1.278    0.213
```

The second model excluding outliers do not strongly changes the results of the model including them. We kept results from the first model including apparent outliers.

3.3.3. Evenness

```
# removing two NAs
Data_Rhopa2<-Data_Rhopa[which(Data_Rhopa$EFtot!="NA"),]
mod_E_Rhopa <- lm(EFtot ~ dPCR300+IFTher300+Flora,
            data = Data_Rhopa2)
```

```
par(mfrow=c(1,3))
plot(mod_E_Rhopa, which=1:2, caption = list("Gaussian", "QQ_plot","", "",""), cex.caption = 0.8)
spatcor_Es<-spline.correlog(x=Data_Rhopa2[, "X"], y=Data_Rhopa2[, "Y"],
z=residuals(mod_E_Rhopa, type="pearson"),na.rm=TRUE, xmax=3000,
resamp=100, npoints=30, quiet = T)
plot(spatcor_Es)
```

a. Variance checking

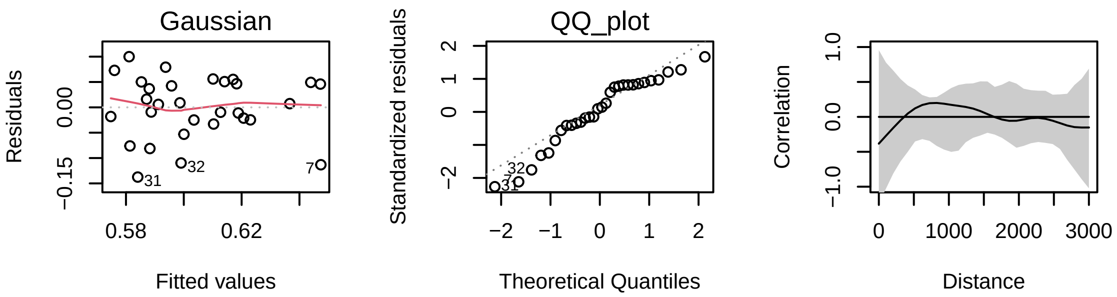

The linear model provides a good fit to the data. No spatial autocorrelation is detected.

```
round(summary(mod_E_Rhopa)$coefficients,3)
```

b. Model coefficients

```
##             Estimate Std. Error t value Pr(>|t|)
## (Intercept)    0.605      0.012  51.958    0.000
## dPCR300        0.008      0.011   0.681    0.502
## IFTher300      0.013      0.012   1.082    0.289
## Flora         -0.013      0.013  -1.054    0.302
```

3.3.4. Community dispersion metric

```
mod_CWMd_Rhopa0 <- lm(CWMd ~ dPCR300+IFTher300+Flora,
            data = Data_Rhopa)
mod_CWMd_Rhopa <- lm(CWMd ~ dPCR300+IFTher300+Flora,
            data = Data_Rhopa, subset=c(-3, -15,-10))
```

```
par(mfrow=c(2,3))
plot(mod_CWMd_Rhopa0, which=1:2, caption = list("Gaussian", "QQ_plot","", "",""), cex.caption = 0.8)
spatcor_Es<-spline.correlog(x=Data_Rhopa[, "X"], y=Data_Rhopa[, "Y"],
z=residuals(mod_CWMd_Rhopa0, type="pearson"),na.rm=TRUE, xmax=3000,
resamp=100, npoints=30, quiet = T)
plot(spatcor_Es)
plot(mod_CWMd_Rhopa, which=1:2, caption = list("Gaussian", "QQ_plot","", "",""), cex.caption = 0.8)
spatcor_Es<-spline.correlog(x=Data_Rhopa[, "X"], y=Data_Rhopa[, "Y"],
z=residuals(mod_CWMd_Rhopa, type="pearson"),na.rm=TRUE, xmax=3000,
resamp=100, npoints=30, quiet = T)
plot(spatcor_Es)
```

a. Variance checking

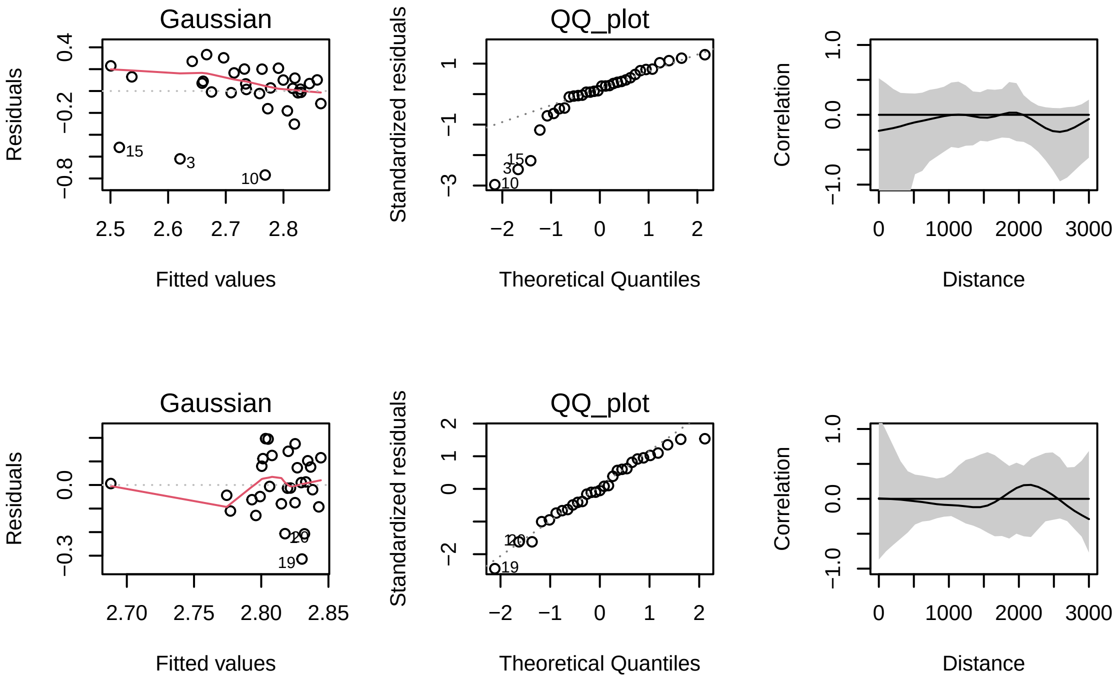

The linear model provides a relative poor fit to the data, due to 3 apparent outliers with excess in negative residual variance (15: site A9; 3: site A11; 10: site A4). Eliminating these outliers ameliorate variance homogeneity. No spatial autocorrelation is detected.

```
round(summary(mod_CWMd_Rhopa0)$coefficients,3)
```

b. Model coefficients

```
##             Estimate Std. Error t value Pr(>|t|)
## (Intercept)    2.737      0.047  58.460    0.000
## dPCR300       -0.016      0.046  -0.348    0.731
## IFTher300     -0.074      0.049  -1.504    0.144
## Flora          0.052      0.051   1.021    0.316
```

```
round(summary(mod_CWMd_Rhopa)$coefficients,3)
```

```
##             Estimate Std. Error t value Pr(>|t|)
## (Intercept)    2.811      0.025 113.319    0.000
## dPCR300       -0.023      0.023  -1.010    0.322
## IFTher300     -0.012      0.027  -0.444    0.661
## Flora          0.013      0.027   0.500    0.622
```

The second model excluding outliers do not strongly changes the results of the model including them. We kept results from the first model including apparent outliers.

3.3.5. Community specialisation metric

```
mod_CWMs_Rhopa <- lm(CWMs ~ dPCR300+IFTher300+Flora,
            data = Data_Rhopa)
```

```
par(mfrow=c(1,3))
plot(mod_CWMs_Rhopa, which=1:2, caption = list("Gaussian", "QQ_plot","", "",""), cex.caption = 0.8)
spatcor_Es<-spline.correlog(x=Data_Rhopa[, "X"], y=Data_Rhopa[, "Y"],
z=residuals(mod_CWMs_Rhopa, type="pearson"),na.rm=TRUE, xmax=3000,
resamp=100, npoints=30, quiet = T)
plot(spatcor_Es)
```

a. Variance checking

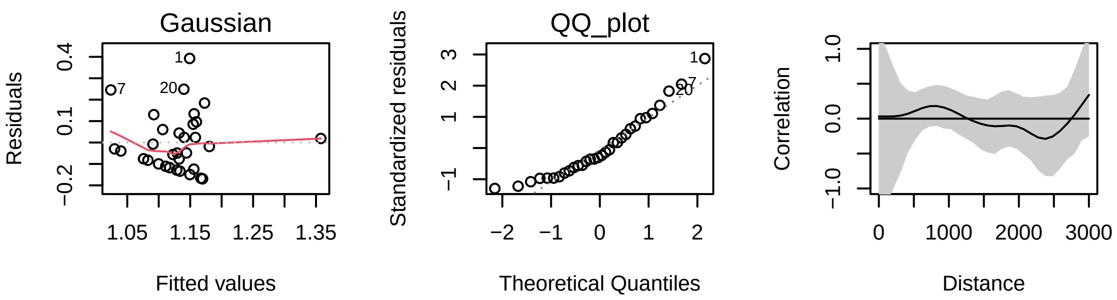

The linear model provides a good fit to the data. No spatial autocorrelation is detected.

```
round(summary(mod_CWMs_Rhopa)$coefficients,3)
```

b. Model coefficients

```
##             Estimate Std. Error t value Pr(>|t|)
## (Intercept)    1.131      0.025  45.095    0.000
## dPCR300        0.035      0.025   1.416    0.168
## IFTher300     -0.032      0.026  -1.190    0.244
## Flora          0.020      0.027   0.736    0.468
```

3.4. Figure (Fig. 4 of the ms)

```
Fig2a<-ggplot(Data_Rhopa, aes(x=dPCR300, y=Abtot)) +
  scale_y_continuous(labels = function(x) round(as.numeric(x), digits=2), limits=c(0,150)) +
  geom_point() + stat_smooth(method="lm", se=T) +
  ylab("") + xlab("Connectivity index (300 m)")+ theme_classic(base_size = 10)+
  ggtitle("b)")+
  theme(plot.title = element_text(size = 10, face = "bold"))+theme_classic(base_size = 10)
Fig2b<-ggplot(Data_Rhopa, aes(x=IFTher300, y=Abtot)) +
  scale_y_continuous(labels = function(x) round(as.numeric(x), digits=2)) +
  geom_point() + stat_smooth(method="lm", se=T) + theme_classic(base_size = 10)+
  ylab(" ") + xlab("Rate of herbicide treatment within 300 m")+
  ggtitle("a)")+
  theme(plot.title = element_text(size = 10, face = "bold"))+theme_classic(base_size = 10)

Fig_2<-grid.arrange(Fig2b,Fig2a, newpage = F, nrow = 2, ncol = 1,
                    left = textGrob("Abundance",rot = 90, vjust = 1.9,
                                    hjust=0.4))
```

```
# Figure 4 of the ms
 ggsave("Thierry_et_al_Fig_4.tiff", width = 8, height = 12, units="cm", dpi=300, Fig_2)
```

4. Data management and analyses for Orthopteran species

4.1 Renaming columns, calculating Evenness, scaling explanatory variables

```
Data_Ortho<-read.table("GLM3_Orthopteres_global_vf.txt", h=T)
Data_Ortho<-Data_Ortho[,c("Site", "X", "Y", "Richesse", "Richesse_pour_evenness",
                          "Diversite_Shannon", "Abondance",
                          "CWM_dispersion","CWM_specialisation","dPC_Orthopteres_300m",
                          "IFT_HERB_min_NA2_100m", "Rec_herbace_bas", "Humidite_num")]

# renommer et numérote les colonnes pour simplifier la lecture des graphiques
colnames(Data_Ortho) <-c( "Site"="Site",                          #1
                          "X"="X",                                #2
                          "Y"="Y",                                #3
                          "Richesse"="RSp",                       #4
                          "Richesse_pour_evenness"="RSpE",        #5
                          "Diversite_Shannon"="Htot",             #6
                          "Abondance"="Abtot",                    #7
                          "CWM_dispersion"="CWMd",                #8
                          "CWM_specialisation"="CWMs",            #9
                          "dPC_Orthopteres_300m"="dPCo300",       #10
                          "IFT_HERB_min_NA2_100m"="IFTher100",    #11
                          "Rec_herbace_bas"="herb_h",             #12
                          "Humidite_num"="Hum")                   #13

# calcul evenness de Pielou - E=H/log2(S)
Data_Ortho$EFtot<-Data_Ortho$Htot/log2(Data_Ortho$RSpE)

# sans les NA (3 valeurs manquantes)
Data_Ortho<-Data_Ortho[which(Data_Ortho$IFTher100!="NA"),]
Data_Ortho<-Data_Ortho[which(Data_Ortho$RSp!="NA"),]
Data_Ortho<-Data_Ortho[which(Data_Ortho$Htot!="NA"),]
Data_Ortho<-Data_Ortho[which(Data_Ortho$herb_h!="NA"),]
# variables explicatives centrées réduites
Data_Ortho$dPCo300<-log(Data_Ortho$dPCo300)
Data_Ortho$dPCo300<-scale(Data_Ortho$dPCo300)
Data_Ortho$IFTher100<-sqrt(Data_Ortho$IFTher100)
Data_Ortho$IFTher100<-scale(Data_Ortho$IFTher100)
Data_Ortho$herb_h<-sqrt(Data_Ortho$herb_h)
Data_Ortho$herb_h<-scale(Data_Ortho$herb_h)
```

```r
print(Data_Ortho)
```

```
##    Site        X       Y RSp RSpE      Htot Abtot     CWMd       CWMs
## 1    A1 614844.9 6876594   4    4 1.2882523    15 3.000000 0.18181818
## 2   A10 611759.1 6874702   6    6 1.5691529    14 3.000000 0.14285714
## 3   A11 611097.9 6874614   7    7 1.7489708    16 2.937500 0.06250000
## 4   A12 613667.2 6874767   5    5 1.4798484    18 3.000000 0.00000000
## 6   A14 612479.5 6875016   5    5 1.4897503    16 3.000000 0.33333333
## 7   A15 613456.2 6875181   7    7 1.5355617    27 2.925926 0.04166667
## 8    A2 614709.6 6876360   7    6 1.4878167    17 3.000000 0.06666667
## 9    A3 614056.8 6876024   5    5 1.5607104     6 3.000000 0.16666667
## 11   A5 613340.1 6875607   3    3 0.8599673    15 3.000000 0.00000000
## 12   A6 614382.3 6876201   7    7 1.3382659    42 3.000000 0.07500000
## 14   A8 612810.2 6875487   4    4 1.2406843    16 3.000000 0.00000000
## 15   A9 612291.7 6874778   6    6 1.5740973    14 3.000000 0.14285714
## 16  C10 617280.6 6877163   7    7 1.8194391    29 2.793103 0.03703704
## 17  C11 618606.9 6878027   7    7 1.0402569    49 3.000000 0.02127660
## 18  C12 618163.2 6877820   7    7 1.3358624    26 2.961538 0.08695652
## 19   C2 617954.0 6876284   4    4 1.2798542    25 3.000000 0.70000000
## 21   C4 617879.8 6876726   2    2 0.6931472    20 3.000000 0.50000000
## 22   C5 617326.8 6876514   3    2 0.6365142    10 2.000000 0.66666667
## 23   C6 617292.0 6876753   3    2 0.5004024    18 2.800000 0.20000000
## 24   C7 617886.3 6877084   3    2 0.4505612    12 2.750000 0.16666667
## 25   C8 618147.5 6877491   5    4 0.8002853    38 2.880000 0.04166667
## 26   C9 617736.0 6877338   3    3 1.0986123    10 3.000000 0.00000000
## 27   LF 615104.1 6875877   5    5 0.8131905    33 2.931034 0.13043478
## 29   P1 617188.1 6875161   2    2 0.5004024     6 3.000000 1.00000000
## 30   P2 616158.3 6874631   2    1 0.0000000    17 3.000000 0.00000000
## 31   P3 615571.0 6874389   3    3 0.8486856    13 2.600000 0.22222222
## 32   P4 614974.6 6874105   3    3 0.6837389    19 3.000000 0.11111111
## 33   P5 615233.6 6874295   2    2 0.2868360    25 3.000000 0.00000000
##        dPCo300   IFTher100     herb_h Hum     EFtot
## 1  -0.62759038 -0.11174849 -0.7759679   3 0.6441262
## 2  -0.80954088  1.33472593 -0.7759679   3 0.6070312
## 3  -0.29737613  0.78456781  0.1594703   3 0.6229960
## 4   0.89396131  1.23284838 -1.5327533   3 0.6373360
## 6  -0.57074971  1.37998038 -0.6058842   2 0.6416005
## 7  -0.28843143  1.92563726 -0.6058842   3 0.5469781
## 8   1.17567228  0.86079155 -0.9976252   3 0.5755661
## 9   0.06262893  0.86740048 -0.3361705   3 0.6721614
## 11  1.07030216  0.03833501 -0.2219625   3 0.5425789
## 12  0.08865693  0.98044459 -0.7759679   3 0.4766999
## 14  1.14630589 -0.96735483 -1.5327533   3 0.6203421
## 15 -1.02841812  1.91084080  0.1594703   3 0.6089440
## 16 -0.16604160 -0.96735483 -0.3361705   2 0.6480973
## 17 -0.61775322  0.24134700  1.3982640   3 0.3705470
## 18 -1.11689881 -0.01454271  1.3982640   3 0.4758438
## 19 -0.65248320 -0.96735483  2.3261103   1 0.6399271
## 21  3.16849903 -0.96735483  1.0336309   2 0.6931472
## 22  1.45259450 -0.96735483  0.1594703   2 0.6365142
## 23  1.03035178 -0.96735483  0.1594703   2 0.5004024
## 24  0.18585758 -0.96735483 -0.6058842   2 0.4505612
## 25 -0.76985961 -0.96735483 -0.7759679   2 0.4001427
```

```
## 26  0.03063071 -0.96735483 -0.3361705   3 0.6931472
## 27 -1.10734898 -0.96735483  0.1594703   2 0.3502221
## 29  0.52533534 -0.96735483  2.2511738   1 0.5004024
## 30 -0.38539102 -0.96735483 -0.3361705   2       NaN
## 31 -0.38460073  0.22365519  0.5397889   2 0.5354610
## 32 -1.05954359 -0.10010998 -0.3361705   2 0.4313912
## 33 -0.94876900  0.05408475  1.1428872   2 0.2868360
```

4.2 Colinearity

```
#correlogramme des variables explicatives
Flora_plot1<-pairs(Data_Ortho[c(10:13)],
                  upper.panel = panel.lines2, lower.panel=panel.cor, digits=2)
```

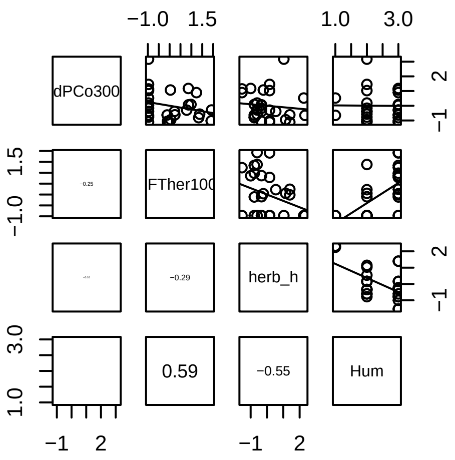


```
pourVIF_Es1<-Data_Ortho[c(10:12)]
corvif(pourVIF_Es1)
```

```
##
##
## Variance inflation factors
##
##               GVIF
## dPCo300   1.105585
## IFTher100 1.196410
## herb_h    1.133743
```

We found negligible collinearities ($r < 0.30$) between the level of herbicide treatment in a 100 m radius around sites, connectivity index (radius 300m), and cover in low height herbaceous species, therefore kept as explanatory variables in statistical analyses. Relative moisture was positively correlated to rate of herbicide use (r = 0.59), and negatively correlated to herbaceous height (r = -0.55), hence was not included as an explanatory variable in statistical analyses.

4.3 Data analysis

4.3.1. Species richness

```
mod_Rsp_Ortho <- lm(RSp ~ dPCo300+IFTher100+herb_h,
            data = Data_Ortho)
```

```
par(mfrow=c(1,3))
plot(mod_Rsp_Ortho, which=1:2, caption = list("Gaussian", "QQ_plot","", "",""), cex.caption = 0.8)
spatcor_Es<-spline.correlog(x=Data_Ortho[, "X"], y=Data_Ortho[, "Y"],
z=residuals(mod_Rsp_Ortho, type="pearson"),na.rm=TRUE, xmax=3000,
resamp=100, npoints=30, quiet = T)
plot(spatcor_Es)
```

a. Variance checking

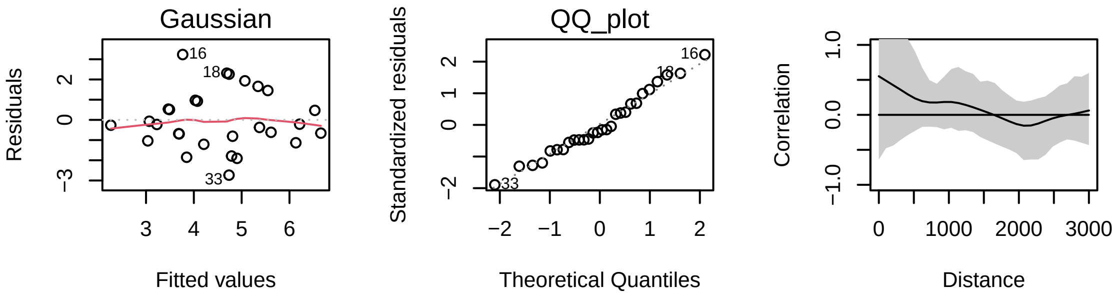

The linear model provides a good fit to the data. No spatial autocorrelation is detected.

```
round(summary(mod_Rsp_Ortho)$coefficients,3)
```

b. Model coefficients

```
##               Estimate Std. Error t value Pr(>|t|)
## (Intercept)      4.536      0.290  15.666    0.000
## dPCo300         -0.377      0.310  -1.215    0.236
## IFTher100        0.922      0.322   2.860    0.009
## herb_h          -0.181      0.314  -0.578    0.569
```

4.3.2. Abundance

```
mod_Abtot_Ortho <- lm(Abtot ~ dPCo300+IFTher100+herb_h,
            data = Data_Ortho)
Data_Ortho1<-Data_Ortho[which(Data_Ortho$Site!="C8"),]
Data_Ortho1<-Data_Ortho1[which(Data_Ortho1$Site!="C11"),]
Data_Ortho1<-Data_Ortho1[which(Data_Ortho1$Site!="A6"),]
mod_Abtot_Ortho1 <- lm(Abtot ~ dPCo300+IFTher100+herb_h,
            data = Data_Ortho1)
```

```
par(mfrow=c(2,3))
plot(mod_Abtot_Ortho, which=1:2, caption = list("Gaussian", "QQ_plot","", "",""), cex.caption = 0.8)
spatcor_Es<-spline.correlog(x=Data_Ortho[, "X"], y=Data_Ortho[, "Y"],
z=residuals(mod_Abtot_Ortho, type="pearson"),na.rm=TRUE, xmax=3000,
resamp=100, npoints=30, quiet = T)
plot(spatcor_Es)
plot(mod_Abtot_Ortho1, which=1:2, caption = list("Without outliers", "","", "",""), cex.caption = 0.8)
spatcor_Es<-spline.correlog(x=Data_Ortho[, "X"], y=Data_Ortho[, "Y"],
z=residuals(mod_Abtot_Ortho1, type="pearson"),na.rm=TRUE, xmax=3000,
resamp=100, npoints=30, quiet = T)
plot(spatcor_Es)
```

a. Variance checking

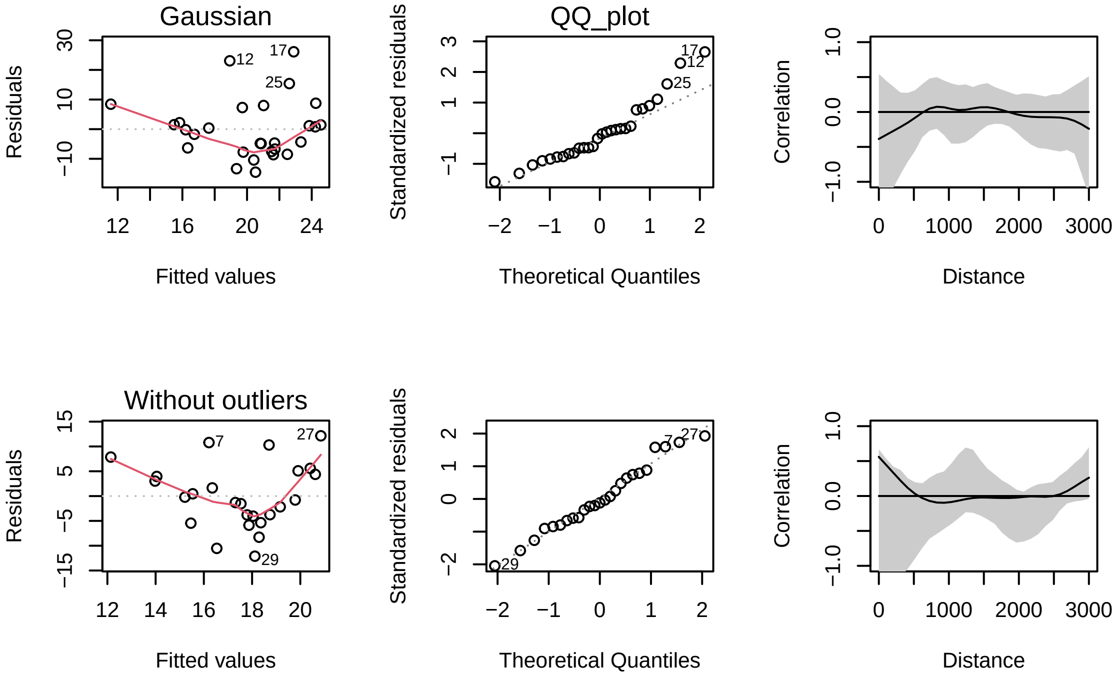

The linear model (Gaussian error variance) has 3 influential points that produce variation heterogeneity through inflated residuals distribution for upper fitted values. Eliminating hese 3 outliers ameliorate model variance homogeneity.No spatial autocorrelation is detected.

```
round(summary(mod_Abtot_Ortho)$coefficients,3)
```

b. Model coefficients

```
##             Estimate Std. Error t value Pr(>|t|)
## (Intercept)   20.214      1.991  10.153    0.000
## dPCo300       -3.095      2.132  -1.452    0.160
## IFTher100     -0.527      2.218  -0.237    0.814
## herb_h         0.634      2.159   0.294    0.772
```

```
round(summary(mod_Abtot_Ortho1)$coefficients,3)
```

```
##             Estimate Std. Error t value Pr(>|t|)
## (Intercept)   17.578      1.389  12.659    0.000
## dPCo300       -2.106      1.457  -1.446    0.163
## IFTher100     -0.919      1.567  -0.586    0.564
## herb_h         0.335      1.544   0.217    0.830
```

The second model excluding outliers do not changes the results of the model including them. We kept results from the first model including apparent outliers.

4.3.3. Evenness

```
mod_E_Ortho <- lm(EFtot ~ dPCo300+IFTher100+herb_h,
                  data = Data_Ortho)
```

```
par(mfrow=c(1,3))
plot(mod_E_Ortho, which=1:2, caption = list("Gaussian", "QQ_plot","", "",""), cex.caption = 0.8)
spatcor_Es<-spline.correlog(x=Data_Ortho[, "X"], y=Data_Ortho[, "Y"],
z=residuals(mod_E_Ortho, type="pearson"),na.rm=TRUE, xmax=3000,
resamp=100, npoints=30, quiet = T)
plot(spatcor_Es)
```

a. Variance checking

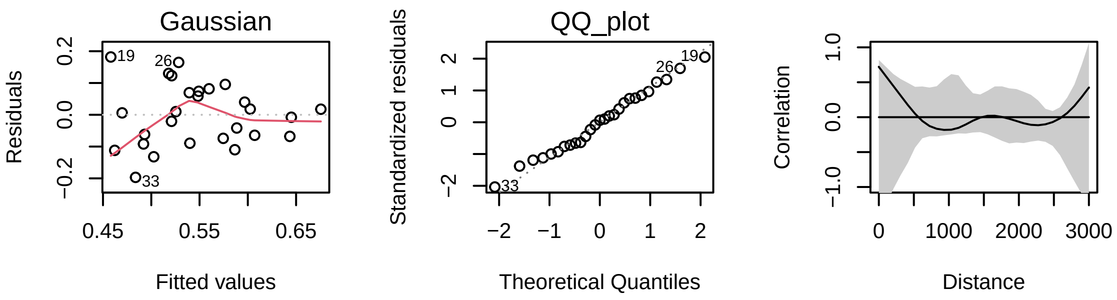

The linear model has 3 influential points that only marginally produce variation heterogeneity through inflated residuals distribution for minimal fitted values - without consequences on residuals distribution.

```
round(summary(mod_E_Ortho)$coefficients,3)
```

b. Model coefficients

```
##             Estimate Std. Error t value Pr(>|t|)
## (Intercept)    0.547      0.020  27.688    0.000
## dPCo300        0.053      0.021   2.503    0.020
## IFTher100      0.025      0.022   1.138    0.267
## herb_h        -0.013      0.021  -0.607    0.550
```

4.3.4. Community specialisation

```
mod_CWMs_Ortho <- lm(CWMs ~ dPCo300+IFTher100+herb_h,
            data = Data_Ortho)
Data_Ortho4<-Data_Ortho[which(Data_Ortho$Site!="P1"),]
Data_Ortho4<-Data_Ortho4[which(Data_Ortho4$Site!="C5"),]
mod_CWMs_Ortho2 <- lm(CWMs ~ dPCo300+IFTher100+herb_h,
            data = Data_Ortho4)
mod_CWMs_Ortho3 <- lm(sqrt(CWMs) ~ dPCo300+IFTher100+herb_h,
            data = Data_Ortho)
```

```
par(mfrow=c(3,3))
plot(mod_CWMs_Ortho, which=1:2, caption = list("Gaussian", "QQ_plot","", "",""), cex.caption = 0.8)
spatcor_Es<-spline.correlog(x=Data_Ortho[, "X"], y=Data_Ortho[, "Y"],
z=residuals(mod_CWMs_Ortho, type="pearson"),na.rm=TRUE, xmax=3000,
resamp=100, npoints=30, quiet = T)
plot(spatcor_Es)
plot(mod_CWMs_Ortho2, which=1:2, caption = list("Gaussian", "QQ_plot","", "",""), cex.caption = 0.8)
spatcor_Es<-spline.correlog(x=Data_Ortho4[, "X"], y=Data_Ortho4[, "Y"],
z=residuals(mod_CWMs_Ortho2, type="pearson"),na.rm=TRUE, xmax=3000,
resamp=100, npoints=30, quiet = T)
plot(spatcor_Es)
plot(mod_CWMs_Ortho3, which=1:2, caption = list("Gaussian (sqrt)", "QQ_plot","", "",""), cex.caption = [illegible]
spatcor_Es<-spline.correlog(x=Data_Ortho[, "X"], y=Data_Ortho[, "Y"],
z=residuals(mod_CWMs_Ortho3, type="pearson"),na.rm=TRUE, xmax=3000,
resamp=100, npoints=30, quiet = T)
plot(spatcor_Es)
```

a. Variance checking

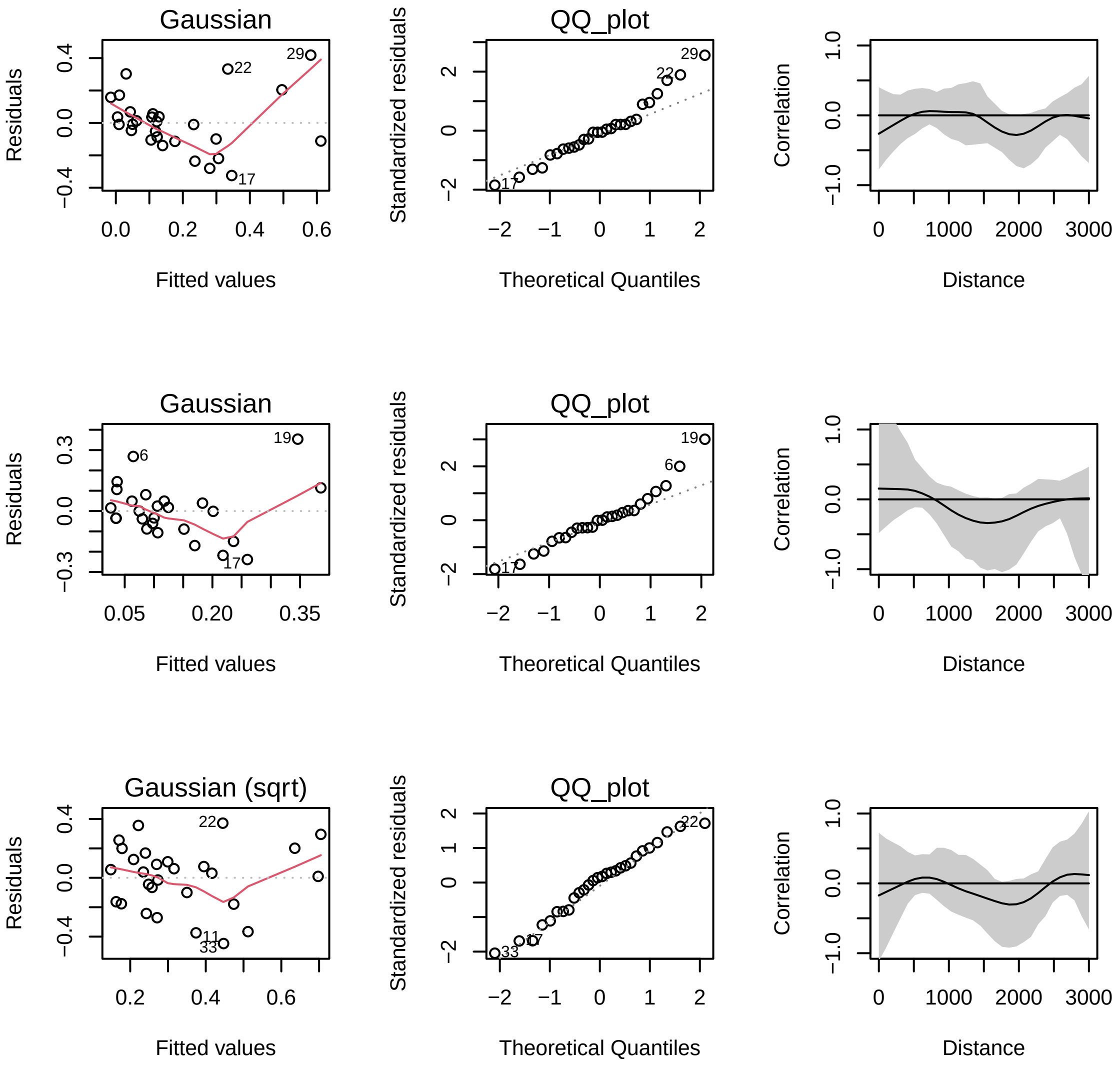

The linear model has 2 influential points that produce variation heterogeneity through inflated residuals distribution for maximal fitted values - with consequences on residuals distribution. Eliminating these outliers do not ameliorate variance homogeneity. Using a lsquare root transformation on the CWMd response variable ameliorates requirements for variance homogeneity.

```
round(summary(mod_CWMs_Ortho)$coefficients,3)
```

b. Model coefficients

```
##             Estimate Std. Error t value Pr(>|t|)
## (Intercept)    0.182      0.036   5.115    0.000
```

```
## dPCo300        0.083      0.038   2.173    0.040
## IFTher100     -0.007      0.040  -0.185    0.855
## herb_h         0.155      0.039   4.017    0.001
```

```
round(summary(mod_CWMs_Ortho2)$coefficients,3)
```

```
##             Estimate Std. Error t value Pr(>|t|)
## (Intercept)    0.144      0.028   5.104    0.000
## dPCo300        0.045      0.030   1.474    0.155
## IFTher100      0.005      0.030   0.175    0.863
## herb_h         0.101      0.032   3.123    0.005
```

```
round(summary(mod_CWMs_Ortho3)$coefficients,3)
```

```
##             Estimate Std. Error t value Pr(>|t|)
## (Intercept)    0.335      0.044   7.642    0.000
## dPCo300        0.068      0.047   1.459    0.158
## IFTher100      0.014      0.049   0.290    0.775
## herb_h         0.154      0.047   3.252    0.003
```

Using the square root transformation on the response variable does not merely changes models interpretation, and gives arguments not to trust any effect of connectivity index on Orthopteran community specialisation metrics.

4.4. Figure (Fig. 5 of the ms)

```
Fig3a<-ggplot(Data_Ortho, aes(x=IFTher100, y=RSp)) +
  geom_point() + stat_smooth(method="lm", se=T) +
  scale_y_continuous(labels = function(x) round(as.numeric(x), digits=2)) +
  ylab("Species richness") + xlab("Rate of herbicide treatment within 100 m")+
  ggtitle("a)")+
  theme(plot.title = element_text(size = 10, face = "bold"))+theme_classic(base_size = 9)
Fig3b<-ggplot(Data_Ortho4, aes(x=dPCo300, y=EFtot)) +
  geom_point() + stat_smooth(method="lm", se=T) +
  scale_y_continuous(labels = function(x) round(as.numeric(x), digits=2)) +
  ylab("Evenness") + xlab("Connectivity index (300 m)")+
  ggtitle("b)")+
  theme(plot.title = element_text(size = 10, face = "bold"))+theme_classic(base_size = 9)
Fig3c<-ggplot(Data_Ortho, aes(x=herb_h, y=CWMs)) +
  geom_point() + stat_smooth(method="lm", se=T) +
  scale_y_continuous(labels = function(x) round(as.numeric(x), digits=2)) +
  ylab("Specialisation") + xlab("Herbaceous cover")+
  ggtitle("c)")+
  theme(plot.title = element_text(size = 10, face = "bold"))+theme_classic(base_size = 9)

Fig_3<-grid.arrange(Fig3a, Fig3b, Fig3c, newpage = F, nrow = 3, ncol = 1)
```

```
# Figure 5 of the ms
 ggsave("Thierry_et_al_Fig_5.tiff", width = 8, height = 18, units="cm", dpi=300, Fig_3)
```

5. Correlations between community diversity measures (Table 4 of the ms)

```
Data_corTOT<-read.table("EV_cor_tot2.txt", h=T)
print(Data_corTOT)
```

```
##    Site        X       Y Soil_F Humidite_num RSp_F Abtot_F   EFtot_F   CWMs_F
## 1    A1 614844.9 6876594  Sandy            3    26     130 0.6485919 17.18266
## 2   A10 611759.1 6874702   Clay            3    21      97 0.6522372 17.60140
## 3   A11 611097.9 6874614  Sandy            3    13      70 0.6320175 19.03911
## 4   A12 613667.2 6874767  Sandy            3    28     131 0.6459333 17.72780
## 5   A13 609876.1 6874610   Clay            3    10      47 0.6135332 18.14083
## 6   A14 612479.5 6875016  Sandy            2    14      84 0.6358659 19.77419
## 7   A15 613456.2 6875181   Clay            3     8      34 0.5771166 18.87681
## 8    A2 614709.6 6876360  Sandy            3    18     109 0.6479895 18.58878
## 9    A3 614056.8 6876024  Sandy            3    14      81 0.6339113 18.63649
## 10   A4 613831.3 6875933  Sandy            3    26     135 0.6448666 17.82707
## 11   A5 613340.1 6875607  Sandy            3    22     101 0.6310626 17.21313
## 12   A6 614382.3 6876201   Clay            3    18      86 0.6315714 17.78569
## 13   A7 613586.1 6875760   Clay            3    11      67 0.6328047 18.68224
## 14   A8 612810.2 6875487   Clay            3    13      92 0.6727504 17.36072
## 15   A9 612291.7 6874778   Clay            3    12      60 0.6116899 18.97475
## 16  C10 617280.6 6877163   Clay            2    21      96 0.6276262 18.67776
## 17  C11 618606.9 6878027   Clay            3    13      79 0.6493669 17.11607
## 18  C12 618163.2 6877820   Clay            3    14      83 0.6470439 18.74913
## 19   C2 617954.0 6876284  Sandy            1    29     191 0.6664842 17.81353
## 20   C3 617907.2 6876382  Sandy            1    28     174 0.6342371 18.17829
## 21   C4 617879.8 6876726  Sandy            2    35     180 0.6523201 17.39880
## 22   C5 617326.8 6876514  Sandy            2    29     136 0.6535928 18.82222
## 23   C6 617292.0 6876753  Sandy            2    17      92 0.6357073 17.36214
## 24   C7 617886.3 6877084  Sandy            2    19     110 0.6669694 17.92448
## 25   C8 618147.5 6877491   Clay            2    26     128 0.6368155 17.86489
## 26   C9 617736.0 6877338   Clay            3    17     121 0.6607976 18.01449
## 27   LF 615104.1 6875877  Sandy            2    26     112 0.6322577 17.34791
## 28   MF 615462.3 6869730   Clay            3    22      97 0.6439520 17.95912
## 29   P1 617188.1 6875161  Sandy            1    30     203 0.6655068 18.94834
## 30   P2 616158.3 6874631  Sandy            2    23     160 0.6579671 18.21086
## 31   P3 615571.0 6874389  Sandy            2    31     193 0.6600572 18.43825
## 32   P4 614974.6 6874105  Sandy            2    22     109 0.6373855 18.85358
## 33   P5 615233.6 6874295  Sandy            2    23     144 0.6563807 18.03600
## 34   V2 605292.9 6873804   Clay            2    16      87 0.6340411 18.08463
## 35  V36 605746.3 6873914   Clay            2    24     111 0.6464674 17.57257
##       CWMd_F  CWMdp_F RSp_R Abtot_R   EFtot_R   CWMs_R   CWMd_R RSp_O Abtot_O
## 1  1.0385385 57.38710     8      24 0.6031827 1.541667 2.625000     4      15
## 2  1.2652747 51.05495     4       8 0.6381250 1.000000 2.750000     6      14
## 3  1.2305263 34.41818     1       1        NA 1.000000 2.000000     7      16
## 4  0.9990698 50.92248     9      23 0.6077220 1.000000 2.913043     5      18
## 5  1.3442553 42.00000     5      10 0.5970445 1.000000 2.900000    NA      NA
## 6  1.2486486 28.72222     3       3 0.6931472 1.000000 2.666667     5      16
## 7  1.2261765 29.17647     9      26 0.5342114 1.269231 2.730769     7      27
## 8  1.2552632 41.50000     5       6 0.6648307 1.000000 2.666667     7      17
## 9  1.0470370 40.55000     4      12 0.5994247 1.083333 2.750000     5       6
```

```
## 10 0.9917164 53.21481     1       1        NA 1.000000 2.000000    NA      NA
## 11 0.8351485 47.11881     7      26 0.5983633 1.000000 2.730769     3      15
## 12 1.1466667 55.56410     5       9 0.6721614 1.222222 3.000000     7      42
## 13 0.9400000 48.96923     4       6 0.6648307 1.166667 3.000000    NA      NA
## 14 0.8756522 37.20652    10      26 0.6030123 1.080000 2.880000     4      16
## 15 0.9896667 27.36667     4       8 0.6931472 1.000000 2.000000     6      14
## 16 1.0759783 43.88043     8      16 0.5773753 1.066667 2.933333     7      29
## 17 0.8967105 51.82857     8      11 0.6810640 1.181818 2.818182     7      49
## 18 0.8756098 46.76923     7      21 0.6729321 1.095238 3.000000     7      26
## 19 0.4773936 44.10526     8      31 0.6247206 1.290323 2.516129     4      25
## 20 0.5617647 43.04094     7      36 0.5469800 1.388889 2.611111    NA      NA
## 21 0.7312048 54.11377     6      86 0.6439776 1.376471 2.694118     2      20
## 22 0.8419828 47.70248     9      69 0.5070646 1.161765 2.808824     3      10
## 23 0.8941772 58.29114     6      68 0.6075647 1.164179 2.805970     3      18
## 24 0.9791919 49.03030     7      75 0.5795153 1.240000 2.840000     3      12
## 25 1.0313386 53.41270    10      34 0.5785512 1.176471 2.735294     5      38
## 26 1.0921818 43.74545     7      40 0.6355140 1.256410 2.846154     3      10
## 27 1.1377778 49.28713    12      28 0.6489380 1.357143 2.750000     5      33
## 28 0.9374725 55.51111    NA      NA        NA       NA       NA    NA      NA
## 29 0.4213441 52.67347     4      10 0.6660895 1.000000 2.800000     2       6
## 30 0.6823448 49.88667     5      47 0.5052627 1.000000 2.913043     2      17
## 31 0.6296216 51.11351     6      32 0.4465416 1.031250 2.937500     3      13
## 32 0.9000971 49.78241     6      54 0.4893391 1.055556 2.962963     3      19
## 33 0.7517730 62.70423     7      27 0.5566488 1.000000 2.960000     2      25
## 34 1.1545455 28.06494    NA      NA        NA       NA       NA    NA      NA
## 35 1.3654545 44.11818    NA      NA        NA       NA       NA    NA      NA
##      EFtot_O     CWMs_O   CWMd_O
## 1  0.6441262 0.18181818 3.000000
## 2  0.6070312 0.14285714 3.000000
## 3  0.6229960 0.06250000 2.937500
## 4  0.6373360 0.00000000 3.000000
## 5         NA         NA       NA
## 6  0.6416005 0.33333333 3.000000
## 7  0.5469781 0.04166667 2.925926
## 8  0.5755661 0.06666667 3.000000
## 9  0.6721614 0.16666667 3.000000
## 10        NA         NA       NA
## 11 0.5425789 0.00000000 3.000000
## 12 0.4766999 0.07500000 3.000000
## 13        NA         NA       NA
## 14 0.6203421 0.00000000 3.000000
## 15 0.6089440 0.14285714 3.000000
## 16 0.6480973 0.03703704 2.793103
## 17 0.3705470 0.02127660 3.000000
## 18 0.4758438 0.08695652 2.961538
## 19 0.6399271 0.70000000 3.000000
## 20        NA         NA       NA
## 21 0.6931472 0.50000000 3.000000
## 22 0.6365142 0.66666667 2.000000
## 23 0.5004024 0.20000000 2.800000
## 24 0.4505612 0.16666667 2.750000
## 25 0.4001427 0.04166667 2.880000
## 26 0.6931472 0.00000000 3.000000
## 27 0.3502221 0.13043478 2.931034
```

```
## 28        NA         NA       NA
## 29 0.5004024 1.00000000 3.000000
## 30        NA 0.00000000 3.000000
## 31 0.5354610 0.22222222 2.600000
## 32 0.4313912 0.11111111 3.000000
## 33 0.2868360 0.00000000 3.000000
## 34        NA         NA       NA
## 35        NA         NA       NA
```

```
Data_corTOT$Soil_Fn<-as.numeric(as.factor(Data_corTOT$Soil_F))
print(cor_plotTOT<-pairs(Data_corTOT[c(5:22)],
                upper.panel = panel.lines2, lower.panel=panel.cor, digits=2))
```

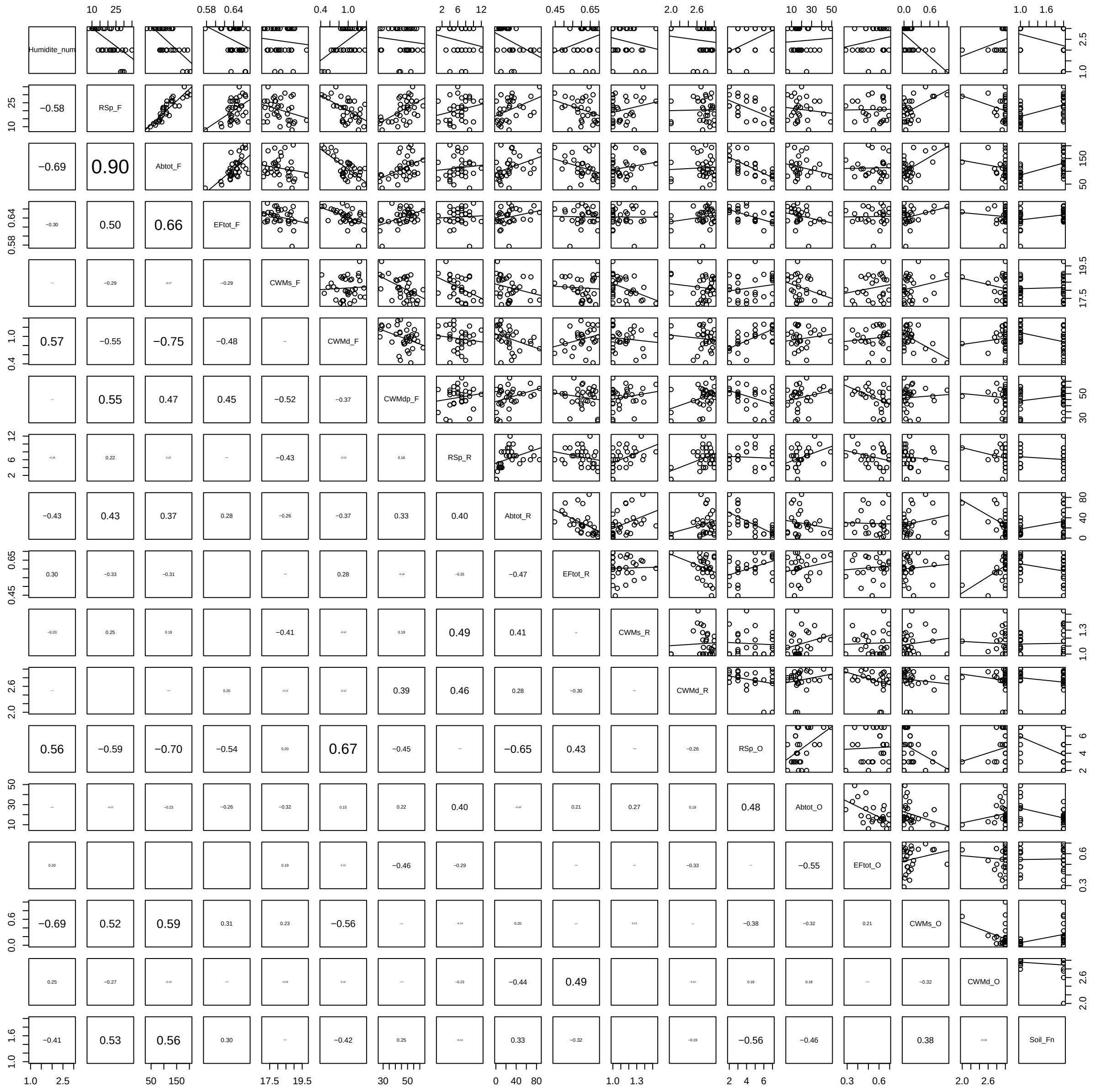

```
## NULL
```
